# Adaptive defence-related changes in the metabolome of *Sorghum bicolor* cells in response to lipopolysaccharides of the pathogen *Burkholderia andropogonis*

**DOI:** 10.1038/s41598-020-64186-y

**Published:** 2020-05-06

**Authors:** Charity R. Mareya, Fidele Tugizimana, Flaviana Di Lorenzo, Alba Silipo, Lizelle A. Piater, Antonio Molinaro, Ian A. Dubery

**Affiliations:** 1 Research Centre for Plant Metabolomics, Department of Biochemistry, University of Johannesburg, Auckland Park, 2006 South Africa; 2 Department of Chemical Sciences, University of Napoli Federico II, Complesso Universitario Monte Sant’Angelo, Via Cintia 4, 80126 Napoli, Italy

**Keywords:** Metabolomics, Metabolomics, Plant immunity, Plant immunity

## Abstract

Plant cell suspension culture systems are valuable for the study of complex biological systems such as inducible defence responses and aspects of plant innate immunity. Perturbations to the cellular metabolome can be investigated using metabolomic approaches in order to reveal the underlying metabolic mechanism of cellular responses. Lipopolysaccharides from the sorghum pathogen, *Burkholderia andropogonis* (LPS_*B.a*._), were purified, chemically characterised and structurally elucidated. The lipid A moiety consists of tetra- and penta-acylated 1,4’-*bis*-phosphorylated disaccharide backbone decorated by aminoarabinose residues, while the O-polysaccharide chain consists of linear trisaccharide repeating units of [→2)-α-Rha3*C*Me-(1 → 3)-α-Rha-(1 → 3)-α-Rha-(1 → ]. The effect of LPS_*B.a*._ in triggering metabolic reprogramming in *Sorghum bicolor* cells were investigated using untargeted metabolomics with liquid chromatography coupled to mass spectrometry detection. Cells were treated with LPS_*B.a*._ and the metabolic changes monitored over a 30 h time period. Alterations in the levels of phytohormones (jasmonates, zeatins, traumatic-, azelaic- and abscisic acid), which marked the onset of defence responses and accumulation of defence-related metabolites, were observed. Phenylpropanoids and indole alkaloids as well as oxylipins that included di- and trihydroxyoctadecedienoic acids were identified as signatory biomarkers, with marked secretion into the extracellular milieu. The study demonstrated that sorghum cells recognise LPS_*B.a*._ as a ‘microbe-associated molecular pattern’, perturbing normal cellular homeostasis. The molecular features of the altered metabolome were associated with phytohormone-responsive metabolomic reconfiguration of primary and secondary metabolites originating from various metabolic pathways, in support of defence and immunity.

## Introduction

Plants are continuously exposed to an array of pathogens which can either be host-specific or can affect a wide range of hosts. Existing crop protection strategies are vulnerable against novel pathogens or pathogens developing increasing resistance against chemical controls^1^. One of the major diseases affecting *Sorghum bicolor*, an economically important grain crop, is bacterial leaf stripe disease caused by *Burkholderia andropogonis*. Recent metabolomics insights revealed aspects of the molecular mechanisms of the *S. bicolor–B. andropogonis* pathophysiological interaction^2^. The results revealed a dynamic and intricate network of the sorghum defence resources towards *B. andropogonis* in launching a heightened defensive capability for disease suppression^2^.

Plants employ several layers of defence to counter potential pathogens^3,4^. These passive (preformed) and active (induced) responses are triggered upon detection of conserved immunogenic motifs, epitopes or ‘patterns’ such as bacterial lipopolysaccharides (LPS), flagellin and fungal ergosterol, β-glucans and chitin^5^. These ‘microbe/pathogen-associated molecular patterns, M/PAMPs’ are perceived via surface-located ‘pattern-recognition receptors, PRRs’ to initiate signal transduction events linked to MAMP-triggered immunity or MTI^6^ that can be acquired in a systemic manner, contributing to broad-spectrum and durable resistance^1^.

LPS, an amphiphilic lipoglycan located in the outermost membrane of Gram-negative bacteria, provides a protective barrier against environmental stresses and is crucial for survival of the bacteria^7,8^. LPSs lies at the interface of the interaction of host plants with potential pathogens, and although structurally conserved, LPSs may differ within and across species to such an extent that a bacterial cell may contain varying LPS chemotypes^9^.

Generally, LPS has a tripartite structure, consisting of functionally distinct components; the O-polysaccharide (OPS) chain and the core oligosaccharide (COS) which is covalently attached to the lipid A (LA) moiety. The OPS-chain is the hydrophilic outermost component of LPS and exposed to the environment^10,11^. It is built up of repeating units of oligosaccharides and displays variation across Gram-negative bacterial species. The OPS acts in protection of the bacterial cells towards hostile surroundings as found *in planta* and is involved in microbial adhesion to host cells^12^. Bacteria possessing an OPS chain are termed *smooth*-type LPS (S-LPS) whereas the ones lacking it are *rough*-type LPS (R-LPS). The COS is located between the OPS-chain and LA moieties, and connected to the LA *via* 3-deoxy-D-*manno*-oct-2-ulosonic acid (Kdo). The LA domain is generally a *bis*-phosphorylated glucosamine disaccharide, varyingly acylated by fatty acids of various lengths embedded in the bacterial outer membrane^13,14^. The LA domain is highly conserved and contributes to LPS stability. However, environmental and growth conditions may alter its structure, thus affecting biological activity of the whole LPS molecule^9,15^.

In the case of plant pathogenic bacteria, knowledge about the structural features of LPSs contributing to M/PAMP activity is minimal^6,8,16,17^. Factors such as adaptation to different habitats and lifestyles, may lead to dynamic remodulation of the LPS structural features, thus contributing to size- and compositional heterogeneity. Differences in LPS structures can also lead to varying results as they may possibly be sensed by different receptors^4,6,8^. While some information about sensing of LA in *Arabidopsis thaliana* has been reported, mechanistic aspects of sensing of the OPS and COS remains unknown^8^ (Supplementary file 1).

In the current study, LPS isolated from *B. andropogonis* (LPS_*B.a*._), was purified and the structures of the LA and OPS moieties elucidated. As a putative M/PAMP, the effect of LPS_*B.a*._ on triggering metabolic reprogramming and the extent to which this occurs, were investigated using a cell suspension culture system^18,19^. Here, sorghum cells were used in combination with a liquid chromatography-mass spectrometry platform for metabolite analysis. The metabolome reflects the ultimate changes in gene expression and, considering that it is highly responsive to changes in enzyme activity and metabolic fluxes^2,20–23^, measurement of dynamic changes of metabolites would reflect on differential and functional features of defence metabolism triggered by LPS perception. Such results would point to metabolic pathways involved in the antimicrobial defences of sorghum, thus contributing to increasing attempts to unravel the biochemical and molecular mechanisms involved in plant-microbe interactions, insights into plant innate immune responses and host-responses to bacterial infection.

## Results

### Compositional and structural analysis of LPS from *Burkholderia andropogonis*

**Figure 1:**
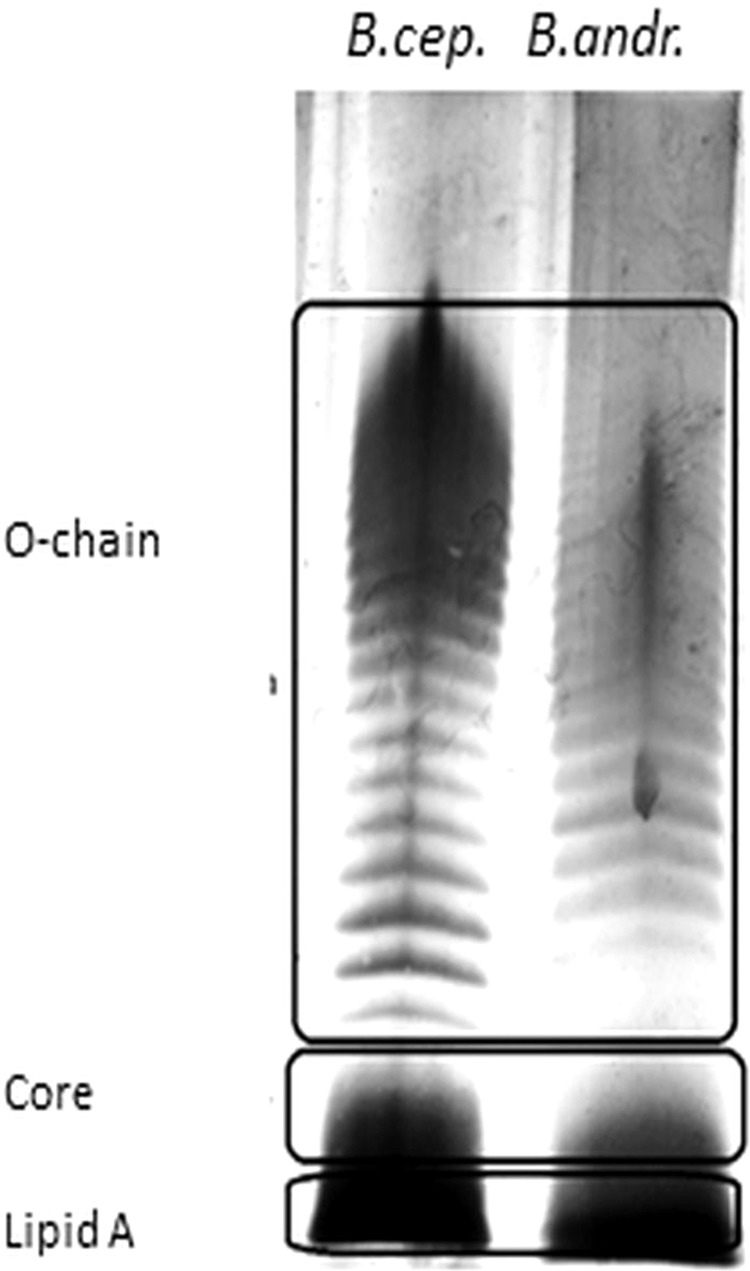
LPS-specific SDS-PAGE analysis of purified LPS_*B.a*._ LPS isolated from *Burkholderia andropogonis* (last lane) is compared to *Burkholderia cepacia* LPS (first lane). LPS_*B.cep*._ was used as a reference as it has been well-characterised^11^. The indicated regions show the three LPS components from the two *Burkholderia* species.

LPS_*B.a*._ consists of a hydrophilic hetero-polysaccharide OPS covalently linked through a COS to the LA of M_r_ approximately 1.5 kDa. An LPS-specific SDS-PAGE analysis of purified LPS_*B.a*._ is shown in Fig. 1, where the LPS displays a characteristic ladder-like pattern indicating the occurrence of a different number of oligosaccharide repeating units that forms the extended OPS.

Compositional analysis of the sugars revealed mainly L-rhamnose (L-Rha) with minor amounts of 2,6-dideoxy-2-amino-D-glucose (D-QuiN), 4-amino-4-deoxy-L-arabinose (L-Ara4N), D-mannose (D-Man), D-galactose (D-Gal), D-glucose (D-Glc), 2-amino-2-deoxy-D-glucose (D-GlcN), L-*glycero*-D-*manno*-heptose (L,D-Hep), 3-deoxy-D-*manno*-oct-2-ulopyranosonic acid (D-Kdo) and D-*glycero*-D-*talo*-oct-2-ulopyranosonic acid (D-Ko). Linkage analysis highlighted the presence of 3-substituted L-Rha and the 2-substituted rhamnose derivative, 3-*C*-methyl-rhamnose (Rha3*C*Me). The absolute configuration of Rha3CMe remains to be determined.

**Figure 2:**
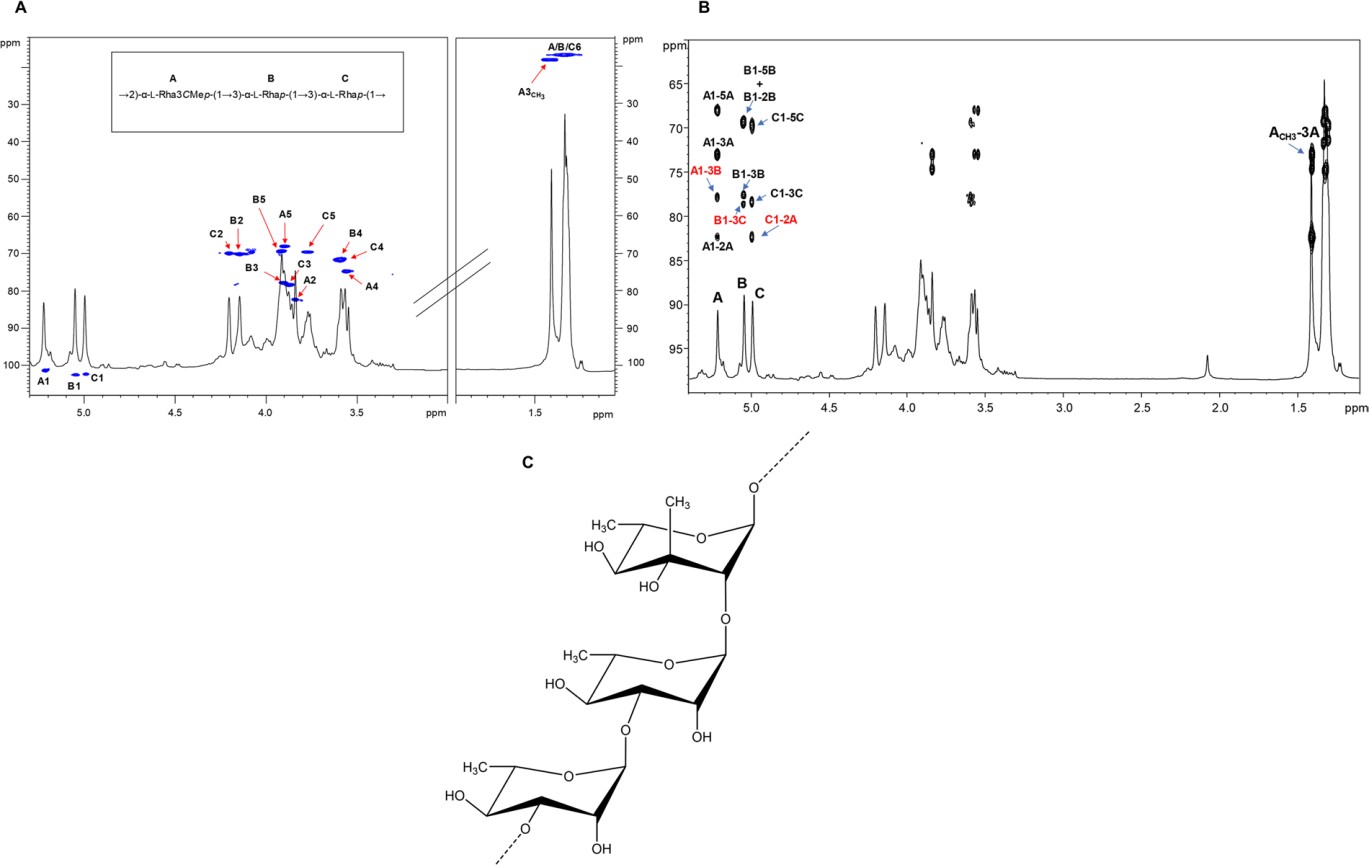
Structural characterisation of the O-polysaccharide (OPS) chain moiety from LPS_*B.a*._ (**A**) Zoom of the overlapped ^1^H and ^1^H, ^13^C HSQC spectra of the OPS from LPS_*B.a*._ after mild acid treatment. The heteronuclear correlations are indicated. Numbering of sugar residues is as reported in Table S1. (**B**) Zoom of the overlapped ^1^H, ^1^H, ^13^C HMBC spectra. The key *inter*-residual long-range correlations involving sugar moieties (**A**–**C**, Table S2) are indicated in red, whereas the *intra*-residual long-range contacts are reported in black; letters are as in Table S1. The one-bond heteronuclear correlation involving the *C*-methyl group of residue **A** is also reported in the spectrum. (**C)** The structure of the elucidated trisaccharide repeating unit of the LPS_*B.a*._ OPS moiety.

To define the OPS moiety of the LPS_*B.a*._, a complete set of 1D and 2D NMR spectroscopy experiments was executed (DQF-COSY, TOCSY, ROESY, NOESY, ^1^H,^13^C HSQC, ^1^H,^13^C HSQC-TOCSY, and ^1^H,^13^C HMBC), revealing a polysaccharide made up of the trisaccharide repeating unit [→2)-α-Rha3*C*Me-(1 → 3)-α-Rha-(1 → 3)-α-Rha-(1 → ]. The ^1^H NMR spectrum for the OPS moiety revealed three main anomeric proton signals detected at *δ*_H_ 5.21, 5.04, and 4.99 ppm relative to three different spin systems and referred to here as **A**, **B**, and **C** (Table S1). The intense methyl signals at *δ*_H_ 1.30, 1.31 ppm and the correlations in the total correlation spectroscopy (TOCSY) spectrum (Fig. S1) agreed with the presence of rhamnose units, as described above, in the sugar compositional analysis on intact LPS_*B.a*._ All sugar units were present as pyranose rings, according to their ^13^C chemical shift values (Table S1, Fig. 2A) and the long-range correlations between C-1/H-1 and H-5/C-5 in the ^13^C,^1^H HMBC spectrum (Fig. 2B, Table S2). The observation of a further signal a *δ*_H_ 1.40 (*δ*_C_ 18.3) corroborated the linkage analysis data relative to the presence of a sugar unit carrying an additional methyl group. Residues **A** (H-1 at 5.21 ppm), **B** (H-1 at 5.04 ppm), and **C** (H-1 at 4.99 ppm) were all identified as α-rhamnose units as demonstrated by the correlations in the TOCSY spectrum, with the methyl group signal resonating at *δ*_H_ 1.30/1.31 ppm, whereas the α-anomeric configuration was defined on the basis of the ^1^*J*_C,H_ values and the *intra*-residual NOE contacts of H-1 with H-2 of each spin system. Finally, the *manno* configuration was confirmed by evaluation of ^3^*J*_H,H_ coupling constant values^10^. Residue **A**, attributed to the Rha3*C*Me (evalose), was distinguished from the Rha residues **B** and **C** by the observation of HMBC correlations of the CH_3_-O group with C-3 at 73.0 ppm (Fig. 2B) as well as by the NOE correlations observed between the methyl group at 1.40 ppm with H-2 and H-5 of the same residue **A**, but not with H-4. This also proved that the methyl group was axial, and consequently that the -OH group at C-3 was equatorial (Fig. 2C).

The down-field shifts of carbon signals identified glycosylated positions at O-2 of **A**, and O-3 of **B** and **C**, in full accordance with the linkage analysis data (Table S1, Fig. 2). The primary sugar sequence composing the OPS repeating unit was determined by analysis of NOESY, ROESY (not shown) and ^1^H, ^13^C HMBC (Fig. 2B) spectra. Briefly, the anomeric signal of residue **A** showed an NOE contact with H-3 of unit **B**, thus indicating a (1 → 3) linkage between **A** and **B**, also confirmed by long-range correlation of H-1 **A** with C-3 of **B** (Fig. 2B). An *inter*-residue NOE correlation was detected between H-1 **B** and H-3 of **C**, whose anomeric proton signal, in turn, presented an NOE contact with H-2 of the Rha3*C*Me **A**. The long-range correlations (Fig. 2B) observed between the anomeric proton of **B** and C-3 of **C** and between H-1 **C** and C-2 of **A** further confirmed the linkages (**B**1 → 3 **C**) and (**C**1 → 2 **A**). In conclusion, by merging NMR and linkage analysis data it was possible to define the complete OPS structure of the LPS_*B.a*._ as a linear trisaccharide repeating unit built up of α−3-*C*-methyl rhamnose spaced by two α-rhamnoses (Fig. 2C).

Investigation of the fatty acids content of the LA revealed the presence of (*R*)−3-hydroxyhexadecanoic acid (C16:0 (3-OH)) with an amide linkage, and of (*R*)−3-hydroxytetradecanoic (C14:0 (3-OH)) and tetradecanoic acid (C14:0) with an ester linkage. The overall chemical composition matched those of the prototype of *Burkholderia* S-LPSs/R-LPSs. Accordingly, the MALDI MS investigation of the LA component, obtained after mild acid hydrolysis of the pure LPS_*B.a*._, showed the occurrence of a mixture of *mono*- and *bis*-phosphorylated LA species (tetra- and penta-acylated) bound by one or two L-Ara4N residues (Fig. S2, Table S3) which is consistent with previously elucidated LA structures from the genus *Burkholderia*^14,24^.

### Non-targeted metabolic profiling of LPS_*B.a.*_-treated cultured sorghum cells

**Figure 3:**
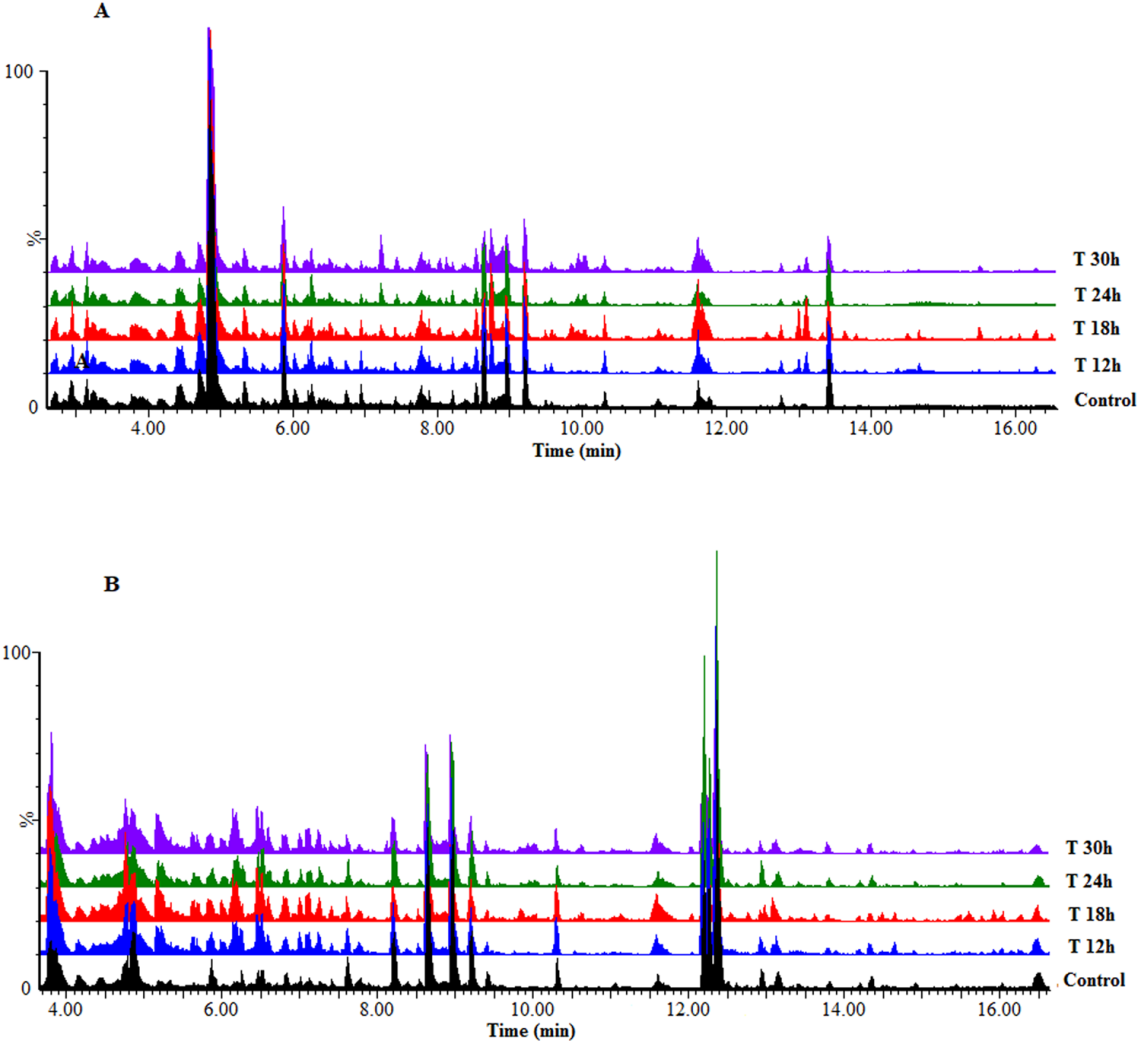
UHPLC-MS BPI chromatograms of methanolic intracellular extracts of sorghum cells treated with LPS_*B.a*._ (**A**): ESI(−) and (**B**): ESI( + ). Representative chromatograms of a control (non-treated 0 h) *vs*. treated samples (12–30 h) display variation related to treatment- and time-related metabolic changes occurring in the cells due to LPS treatment.

Metabolomics deals with the documentation and assessment of altered metabolome states and here ultra-high performance liquid chromatography – electrospray ionisation – quadrupole time-of-flight –high-definition mass spectrometry (UHPLC-ESI-QTOF-HDMS) was employed for the analysis of the endo- and exometabolomes of sorghum cells responding to LPS_*B.a*._ elicitation. Differences with regard to peak population and intensities across treated and non-treated cell extracts were visualised on MS chromatograms obtained from cell extracts (Fig. 3) and growth medium (Fig. S3). Thus, base peak intensity (BPI) chromatograms of these extracts displayed treatment- and time-related metabolic responses. The differences between control *vs*. treated samples, and those across the time points subsequently provided a visual picture of metabolic changes occurring due to the LPS_*B.a*._ treatment as a function of time.

**Figure 4:**
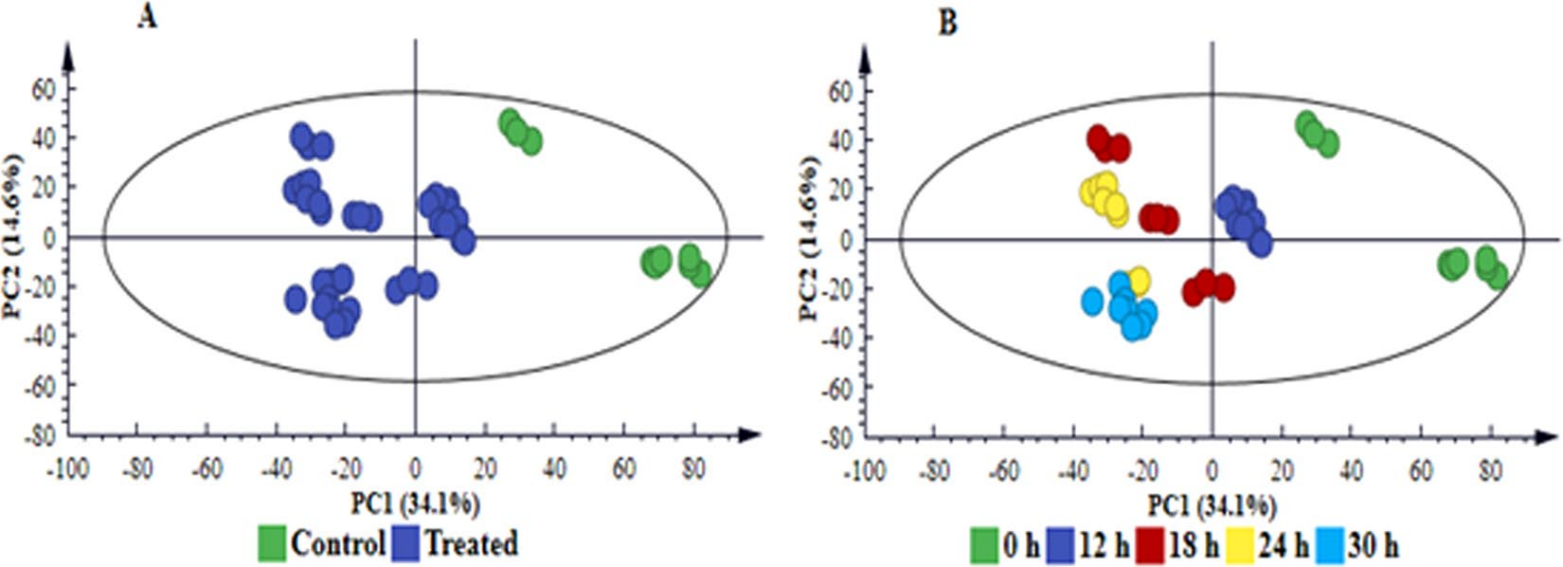
PC analyses of the LC-MS ESI(−) data for intracellular sorghum cell extracts. The 4-component model explains 61.9% variation in Pareto-scaled data, X, and the amount of predicted variation by the model, according to 7-fold cross-validation, is 51.4%. The 7-fold CV procedure is described in the experimental section. (**A)** Clusters coloured based on condition *i.e*. non-treated *vs*. treated shows clear separation between treated and control (non-treated, 0 h) samples. (**B)** is the same scores plot but coloured according to time and showing a clear sequential time trend clustering (from C0 h and T12–30 h). The related model for ESI( + ) data is supplied as Fig. S4, n = 9.

**Figure 5:**
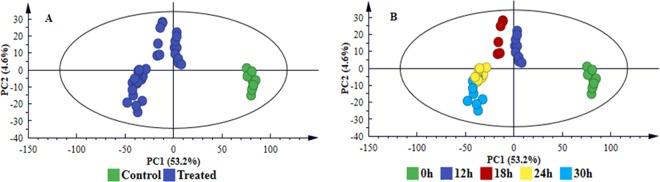
PC analyses of the LC-MS ESI(−) data for extracellular sorghum cell extracts. The 3-component model explains 61.5% variation in Pareto-scaled data, X, and the amount of predicted variation by the model, according to 7-fold cross-validation, is 52.8%. The first 2 PCs were used to generate the above scores plot of all data. Clusters are coloured based on condition *i.e*. non-treated *vs*. treated and show clear separation between treated and control (non-treated, 0 h) samples **(A)** and according to time **(B)**. The related model for ESI( + ) data is supplied as Fig. S5, n = 9.

Multivariate statistical analyses (both unsupervised and supervised) were employed to mine the data and further investigate the observed treatment- and time-related differences. Thorough model validation was consistently applied as described in the experimental section; and only statistically valid models were retained and used. The unsupervised modelling, principal component analysis (PCA), permitted dimension reduction of the data and recognition of groupings, trends and outliers^25,26^. The computed PCA scores plot of cell extracts (Fig. 4 and S4) and medium extracts **(**Fig. 5 and S5**)**, displayed a distinct separation between control and treated samples, revealing treatment-related sample clustering. Colour-coding based on time points showed a distinct sequential time clustering trend of samples, *i.e*. a time-related clustering for both extracts from cells (Fig. 4B and S4B) and medium **(**Fig. 5B and S5B). The clustering of samples highlighted in the PCA scores space, points to differential metabolic changes in cultured sorghum cells in response to LPS_*B.a*._ treatment.

**Figure 6:**
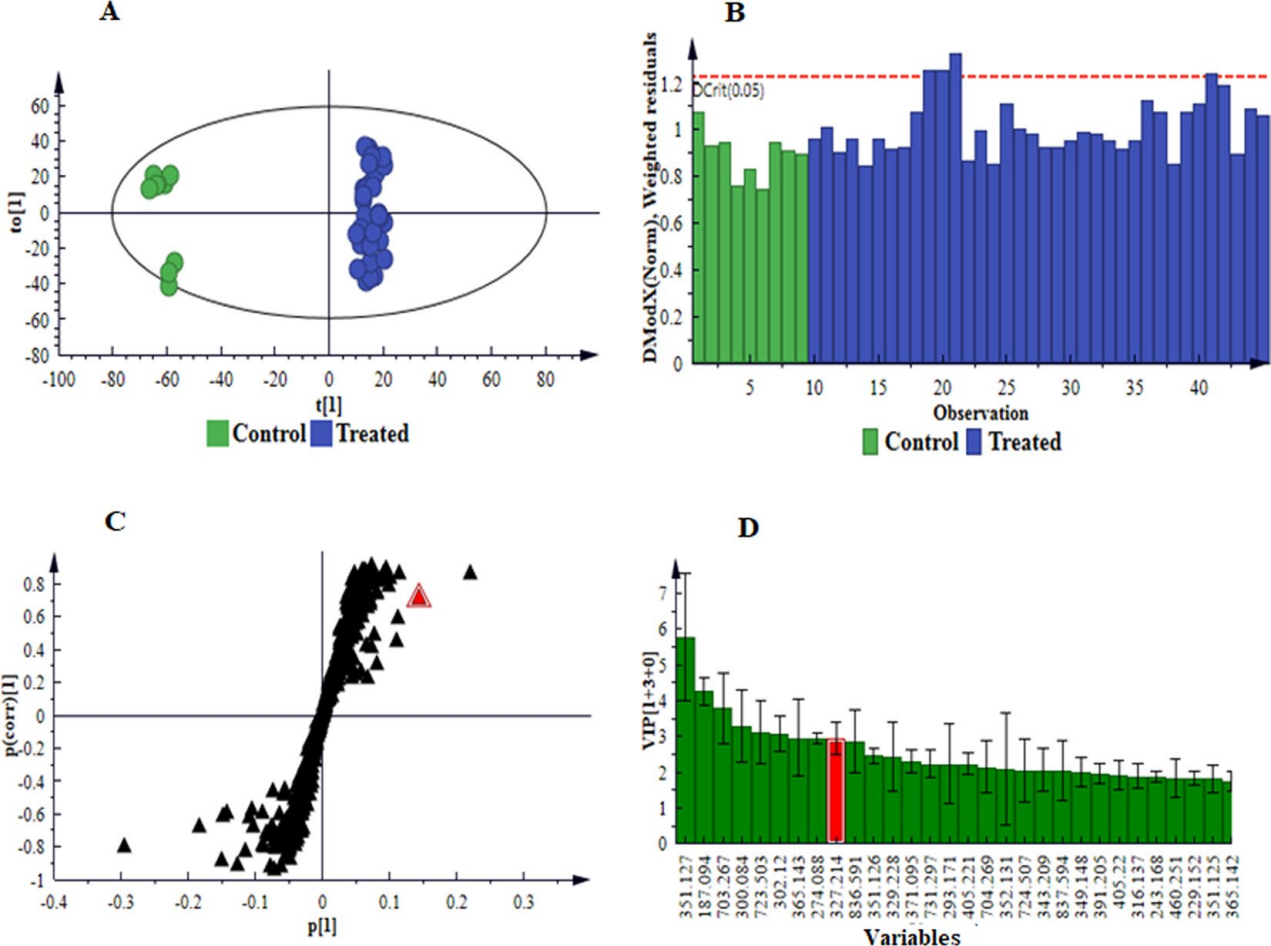
Supervised multivariate analyses of the LC-MS ESI(−) data for intracellular extracts. (**A**) Grouping of control (C 0 h) *vs*. treated (all time points combined) as indicated by an OPLS-DA score plot, n = 9 for each time point. This model comprises 1 predictive component and 3 orthogonal components (R^2^X = 60.0%, R^2^Y = 99.2% and Q^2^ = 95.1%). **(B)** A distance to the model in space X (DModX) plot to detect and evaluate moderate outliers (above the dashed red line, Dcrit). **(C)** An OPLS-DA loadings S-plot displaying the discriminating features (ions) that explain the clustering (sample grouping) observed in the OPLS-DA scores plot, with the features in the top right quadrant positively correlated to the treatment and those in the bottom left quadrant negatively correlated to the treatment. For instance, the selected *m/z* 327.2135 (annotated as trihydroxy-octadecadienoic acid II, Table 1) is positively correlated to the treatment. **(D)** A VIP plot summarising the importance of some of the variables in the projection of the model with the *m/z* values and jackknife confidence intervals reflecting the variable stability. A VIP value >1 indicates a significant variable in the complex analysis in comparing the difference between groups. For instance, the *m/z* 327.2135 (displayed as 327.214), selected from S-plot (**C**), shows a VIP score > 3, indicative of its importance to the model (contributing to the class separation). The equivalent set of graphs for ESI( + ) data is presented as Fig. S6, and in the case of extracellular extracts as Figs. S7: ESI(−) and S8**:** ESI( + ).

For further characterisation and interpretation of the different clustering depicted in PC analyses, a supervised method, orthogonal projection to latent structures analysis (OPLS-DA), was used. The supervised modelling allowed identification of signatory biomarkers underlying the discrimination between the sample classes associated with the LPS treatment. Computed OPLS-DA scores plots showed clear discrimination between the control and treated samples (Fig. 6A and S6A-S8A). Evaluation of the goodness-of-fit (R^2^X(*cum*)), proportion of variance of the response variable explained by the model (R^2^Y(*cum*)) and predictive ability (Q^2^(*cum*)) parameters, indicated that the models were statistically reliable. Further validation also revealed the reliability of the models *i.e*. CV-ANOVA *p*-value of ≤ 0.05^27,28^. In addition, ‘distance to the model in space X’ (DModX) plots (Fig. 6B and S6B-S8B) for the generated OPLS-DA scores plots were used to assess moderate outliers.

The OPLS-DA loadings S-plots (Fig. 6C and S6C-S8C) assisted in visualisation of the covariance and correlation between variables of modelled classes, and permitted the extraction of statistically significant features within the │p[1]│ ≥ 0.05 and │p(corr)│ ≥ 0.5 defined regions, responsible for discrimination between control and LPS_*B.a*._-treated samples. The significance of the extracted variables towards discrimination of samples was assessed using the ‘variable importance in projection’ (VIP) plots (Fig. 6D and S6D-S8D). Only variables with a VIP score> 1^27,29^ were considered significant and selected for further annotation.

Variables relating to the observed metabolic changes due to LPS_*B.a*._ elicitation, selected and validated with the aid of chemometrics tools mentioned above, were putatively identified (at Metabolomics Standards Initiative (MSI) level 2)^30^. Metabolites shown in Table 1 were annotated from both LC-MS ESI( − / + ) data, and had a VIP score > 1. Fold changes presented in Table 1 were obtained from the computed model of C 0 h *vs*. T 18 h, as this was the best time point representation of the overall metabolic changes. Equivalent tables expressing the fold changes and *p*-values generated from the computed OPLS-DA models of other time points (*i.e*. C 0 h *vs*. 12-, 24- and 30 h), for both intra- and extracellular data, are included in the supporting information data file Tables S4 and S5, respectively.

**Table 1:** Annotated discriminatory metabolites from cell (intracellular) and medium (extracellular) extracts of *Sorghum bicolor* cultured cells treated with LPS_*B.a*._ All the metabolites had a VIP score> 1. Fold changes were obtained from OPLS-DA models of control (C0 h) *vs*. treated 18 h. (*Similar data from 12-, 24- and 30 h time points is presented as Supplementary Information*, Tables S4 and S5).

Metabolites	*m/z*	Rt (min)	Adduct	Ion mode	Formula	Intracellular			Extracellular			Class
						*p*-value	Fold change	Trend	*p*-value	Fold change	Trend	
L-Phenylalanine	164.0686	1.84	[M-H]^-^	neg	C_9_H_11_NO_2_	6.28E-06	1.4	Increase	3.07E-11	2.7	Increase	Amino acid
L-Tryptophan	203.0798	2.78	[M-H]^-^	neg	C_11_H_12_N_2_O_2_	2.04E-06	1.4	Increase	0.001	1.4	Increase	Amino acid
16-Hydroxypalmitate	273.2553	13.65	[M + H]^+^	pos	C_16_H_32_O_3_	0.490	1.3	Increase	•	•	•	Fatty acid
15-Hydroxylinoleic acid	295.2253	14.29	[M-H]^-^	neg	C_18_H_32_O_3_	0.001	2.1	Increase	•	•	•	Fatty acid
Dihydroxy-octadecadienoic acid	311.2242	11.79	[M-H]^-^	neg	C_18_H_32_O_4_	1.28E-10	10.8	Increase	0.110	5.2	Increase	Fatty acid
9,10-Dihydroxy-12-octadecenoic acid	313.2354	12.67	[M-H]^-^	neg	C_18_H_34_O_4_	0.606	1.6	Increase	•	•	•	Fatty acid
9,10-Dihydroxystearic acid	315.2511	13.51	[M-H]^-^	neg	C_18_H_36_O_4_	2.37E-09	6.4	Increase	•	•	•	Fatty acid
Trihydroxy-octadecadienoic acid І	327.2149	9.72	[M-H]^-^	neg	C_18_H_32_O_5_	0.377	113	Increase	•	•	•	Fatty acid
Trihydroxy-octadecadienoic acid ІІ	327.2135	11.05	[M-H]^-^	neg	C_18_H_32_O_5_	3.97E-13	356.7	Increase	0.000	38.8	Increase	Fatty acid
9,12,13-Trihydroxy-10-octadecenoic acid	329.2327	9.60	[M-H]^-^	neg	C_18_H_34_O_5_	0.000	1.8	Increase	1.13E-08	9.2	Increase	Fatty acid
Sophoraflavanone G	423.1821	4.42	[M-H]^-^	neg	C_25_H_28_O_6_	2.36E-05	0.8	Decrease	0.030	1.1	Increase	Flavonoid
Apigenin-8-C-glucoside (vitexin)	431.0974	5.58	[M-H]^-^	neg	C_21_H_20_O_10_	0.702	1.1	Increase	0.537	1.2	Increase	Flavonoid
Apigenin-6-C-xyloside-8-C-glucoside (vicenin-1)	565.1545	4.94	[M + H]^+^	pos	C_26_H_28_O_14_	0.064	0.6	Decrease	0.004	1.4	Increase	Flavonoid
Apigenin-6,8-di-C-glucoside(vicenin-2)	595.1687	4.77	[M + H]^+^	pos	C_27_H_30_O_15_	0.082	0.4	Decrease	0.240	2.1	Increase	Flavonoid
Apigenin 7,4’-dimethyl ether	316.1157	8.29	[M + H_NH_3_]^+^	pos	C_17_H_14_O_5_	0.000	0.5	Decrease	•	•	•	Flavonoid
3’,4’5-Trihydroxy-3,7-dimethoxyflavone	367.0221	3.90	[M-H]^-^	neg	C_17_H_20_O_9_	0.032	0.8	Decrease	•	•	•	Flavonoid
4-Coumaroyl-3-hydroxyagmatine	291.1471	5.72	[M-H]^-^	neg	C_14_H_20_N_4_O_3_	0.013	0.7	Decrease	0.001	2.5	Increase	HCA
4-Coumaroylquinic acid	337.1474	1.77	[M-H]^-^	neg	C_16_H_18_O_8_	7.19E-09	0.6	Decrease	0.010	1.1	Increase	HCA
Cinnamoylserotonin	351.1251	2.43	[M-H_HCOOH]^-^	neg	C_19_H_18_N_2_O_2_	7.77E-05	0.8	Decrease	0.000	1.2	Increase	HCA
Feruloylserotonin	351.1266	2.86	[M-H]^-^	neg	C_20_H_20_N_2_O_4_	0.387	0.4	Decrease	0.771	1.2	Increase	HCA
Sinapaldehyde glucoside	369.1199	3.61	[M-H]^-^	neg	C_17_H_22_O_9_	0.525	0.9	Decrease	1.68E-06	2.7	Increase	HCA
1-O-Coumaroyl-beta-D-glucose	371.0957	4.94	[M-H_HCOOH]^-^	neg	C_15_H_18_O_8_	5.26E-11	0.6	Decrease	7.70E-06	2.1	Increase	HCA
Sinapoyl alcohol	209.0764	6.72	[M-H]^-^	neg	C_11_H_14_O_4_	1.55E-10	4.8	Increase	1.34E-05	1.8	Increase	HCA
Dihydroconiferyl alcohol glucoside	413.1422	3.27	[M + H_HCOONa]^+^	pos	C_16_H_24_O_8_	0.005	0.6	Decrease	•	•	•	HCA
Indole-3-butyric acid	272.0893	2.84	[M + H_HCOONa]^+^	pos	C_12_H_13_NO_2_	1.62E-05	0.5	Decrease	•	•	•	Indole compound
N(6)-[(Indol-3-yl)acetyl]-L-lysine	304.1667	4.20	[M + H]^+^	pos	C_16_H_21_N_3_O_3_	1.43E-07	5.8	Increase	•	•	•	Indole compound
Indole-3-acetyl-myo-inositol	353.1348	2.44	[M-H_NH_3_]^-^	neg	C_16_H_19_NO_7_	0.479	1.8	Increase	0.000	2.9	Increase	Indole compound
Indole-3-acetyl-beta-1-D-glucoside	382.1121	3.93	[M-H_HCOOH]^-^	neg	C_16_H_19_NO_7_	0.075	0.7	Decrease	0.925	1.0	Increase	Indole compound
6-Hydroxy-indole-3-acetyl-valine	291.1294	3.89	[M + H]^+^	pos	C_15_H_17_N_2_O_4_	0.174	0.5	Decrease	0.098	2.1	Increase	Indole compound
Traumatic acid	297.1291	3.90	[M + H_HCOONa]^+^	pos	C_12_H_20_O_4_	0.027	4.4	Increase	•	•	•	Phytohormone
(9 R,13 R)−1a,1b-Dihomo-jasmonic acid	239.1638	12.19	[M + H]^+^	pos	C_14_H_22_O_3_	0.016	0.8	Decrease	•	•	•	Phytohormone
Zeatin-7-beta-D-glucoside	397.1826	6.73	[M-H_NH_3_]^-^	neg	C_16_H_23_N_5_O_6_	0.216	0.8	Decrease	•	•	•	Phytohormone
Zeatin	220.1197	2.15	[M + H]^+^	pos	C_10_H_13_N_5_O	2.99E-05	0.5	Decrease	•	•	•	Phytohormone
Methyl jasmonate	247.1298	2.52	[M + H_Na]^+^	pos	C_13_H_20_O_3_	1.21E-08	4.2	Increase	•	•	•	Phytohormone
Dihydrozeatin riboside	354.1769	6.20	[M + H]^+^	pos	C_15_H_23_N_5_O_5_	0.000	0.2	Decrease	•	•	•	Phytohormone
Zeatin riboside	374.1463	5.39	[M + H_Na]^+^	pos	C_15_H_21_N_5_O_5_	0.000	0.3	Decrease	•	•	•	Phytohormone
Azelaic acid	187.0935	6.74	[M-H]^-^	neg	C_9_H_16_O_4_	7.24E-12	6.5	Increase	5.58E-07	1.9	Increase	Phytohormone
Abscisic acid	265.1552	3.35	[M + H]^+^	pos	C_15_H_20_O_4_	7.62E-08	3.5	Increase	4.39E-13	16.8	Increase	Phytohormone
Agmatine	173.0787	5.52	[M-H_NaNa]^-^	neg	C_5_H_14_N_4_	0.000	1.4	Increase	•	•	•	Polyamine
Riboflavin	377.1476	4.49	[M + H]^+^	pos	C_17_H_20_N_4_O_6_	0.050	0.7	Decrease	7.65E-08	5.9	Increase	Flavin

• indicates the metabolite is absent in the particular extract. HCA = hydroxycinnamic acid. Metabolite annotation was achieved with the aid of the Taverna workbench (www.taverna.org.uk), databases such as Dictionary of Natural Products (DNP) (dnp.chemnetbase.com), ChemSpider (www.chemspider.com), PubChem (www.pubchem.ncbi.nlm.nih.gov), PlantCyc (www.plantcyc.org), SorgCyc (www.sorgcyc.org) and KNApSAcK (http://kanaya.naist.jp/knapsack_jsp/top.htm) and available literature^2,23,85^.

*p*-value refers to significance level of a metabolite. Fold change was calculated by dividing the average of the metabolite intensity in replicate samples of treated by the average of the metabolite intensity in replicate samples of control, a value ≥1 represents an increase (metabolite is higher in the treated samples than in the control) and value <1 represents a decrease (metabolite is higher in the control than in the treatment which led to decrease in levels).

## Discussion

### Structural features of the LPS from *B. andropogonis*

As complex amphiphilic lipoglycan macromolecules, LPSs can potentially contain MAMP structures within the OPS, COS and LA moieties^7^. Gram-negative bacteria have developed varied LPSs that can differ in composition. Not only the OPS, but also the bound COS and the LA region can exhibit structural variation, possibly as an adaptive mechanism to hostile environments^9,11^. The OPS chains, projecting from the cell surface of the bacteria are exposed to the environment and host defence systems. The structural variation of LPSs from pathogens and symbionts might be determining factors in the interactions of plants with microbes^31^. For example, the composition, size and structure of the OPS might be dependable indicators of virulence potential. As such, it appears to partake in the molecular communication between host plants and bacteria. With regards to the LA, various structural features were reported to play determining roles in the pathophysiological activity in animals. These include the quantity and position of acyl chains, the overall charge of the LA molecule as affected by the phosphorylation of the disaccharide backbone, and the presence/absence of additional polar headgroups^9,11^. Furthermore, the three-dimensional shape of LA, which in turn is a function of the structural features, has been linked to the differential biological activities of LPSs. Hexaacyl asymmetrical LA exhibits a conical conformation; pentaacyl LA has an intermediary form, and a cylindrical shape is associated with tetraacyl symmetrical LA^32^. In addition to the chemical composition, the structure and conformation of LA are thus central contributory factors during bacterial pathogenesis. Further interest in LA derives from the findings that bacteria are able to regulate the composition of their LA in response to environmental signals, thus modulating or even antagonizing the triggering of host defences^9,11^. Due to the restricted number of elucidated LA structures determined from plant-associated bacteria, no clear relationship between the structure and activity is currently known.

For the LPS from *B. andropogonis*, repeating component of the OPS was characterised as [→2)-α-Rha3*C*Me-(1 → 3)-α-Rha-(1 → 3)-α-Rha-(1 → ]. While rhamnose-containing OPSs in plant-associated bacteria is common, the occurrence of 3-*C*-methylrhamnose (evalose) is rare^33^. Both rhamnose and, particularly its methylated derivative provide highly lipophilic properties to the OPS of *B. andropogonis* which might play a role in the interaction between the bacterium and sorghum cells. Structural analysis on the LA component indicated a heterogeneous tetra- and penta-acylated, 1,4’-*bis*-phosphorylated glucosamine disaccharide backbone, further substituted by L-Ara4N. Analysis of the fatty acids revealed (*R*)−3-hydroxyhexadecanoic acid (16:0(3-OH)), (*R*)−3-hydroxytetradecanoic acid (14:0(3-OH)) and tetradecanoic acid (14:0), matching with the archetypal LA from *Burkholderia* genus. Potentially mimicking what happens in animals, such an under-acylation of the LA may be an important factor likely masking the bacterium from the plant immune system and thus favouring the spread of the infection^34^.

Previously, in a transcriptome investigation of the effect of LPS from *B. cepacia* on *A. thaliana* seedlings, we found that the LA and OPS-COS moieties were active in up-regulating subgroups of genes linked to defence over the same range of gene ontology categories as intact LPS^11^. However, the up-regulation observed in response to intact LPS was more wide-ranging. This suggests that although the molecular patterns of the LA and glycan moieties act as partial agonists, the intact LPS structure is necessary for full agonist activity^11,14^. Since the perception of individual MAMP-active moieties of LPS_*B.a*._ is not yet known, intact LPS_*B.a*._ was used for further investigation. The biological or immuno-modulatory activity of this LPS from the disease-causing *B. andropogonis* was studied by monitoring its ability to lead to reprogramming of the metabolome of sorghum cells.

### Metabolic changes induced by LPS_*B.a.*_ in *Sorghum bicolor* cells

The use of plant cell suspension culture systems as experimental models offers distinct advantages such as short growth cycles, rapid multiplication of a homogeneous population of cells, reduced complexity, decreased biological variability and improved experimental reproducibility. Plant cell suspensions are therefore highly suitable for metabolomics studies^18,19^, and depending on the cell line, ideal for studies on secondary metabolite biosynthetic pathways and inducible defence responses^20–22^.

To gain biological insights into the changes occurring in the sorghum cell culture system following LPS_*B.a*._ perception, the chemometrically selected variables were structurally annotated (Table 1). The endometabolome (intracellular/fingerprint) and exometabolome (extracellular/footprint) of the cells were characterised by metabolites associated with primary as well as secondary metabolism, and of diverse biochemical functions in plant defence. To have a comprehensive picture of metabolic reprogramming in sorghum cells induced by LPS treatment, relative quantification (expressed in fold changes) of the putatively identified metabolites was carried out, enabling a quantitative description of metabolic changes. Metabolomic analyses of the cultured cells system revealed the metabolic reprogramming in *S. bicolor* suspensions triggered by LPS_*B.a*._ to be similar to that in plant leaf tissue, triggered by the live pathogen (*i.e*. similar metabolite classes, that include amino acids, fatty acids, hydroxycinnamic acids, flavonoids, indoles and phytohormones)^2^. However, there were qualitative and quantitative differences in the levels of signatory metabolites identified from the plant *vs*. cell metabolomes. Nonetheless, the similarity in the induced metabolic profiles indicate that the cell suspension culture system is suitable for future research on this plant: microbe interaction.

LPS_*B.a*._ triggered an alteration of several plant hormones (jasmonates, zeatins, traumatic-, azelaic- and abscisic acid). The derivative of jasmonic acid (JA), methyl jasmonic acid (MeJA), accumulated in the intracellular milieu to significantly high levels (Table 1). As a well-known volatile plant stress hormone and one of the major players in induced systemic resistance (ISR) research, this molecule is involved in the activation of defence mechanisms such as programmed cell death (PCD), reactive oxygen species (ROS) production, lignin formation and stimulation of deposition of wax layers in plant tissues^35–37^. MeJA has been reported as a signalling molecule in elicitor-induced plant cell cultures and plant tissue, initiating secondary metabolite accumulation^37,38^. Studies on exogenous application of the hormone, revealed stimulation of phenylpropanoid, flavonoid, fatty acid and other secondary metabolic pathways^28,35^. A study on transcriptional profiling of genes induced by salicylic acid (SA) and MeJA in sorghum revealed that the hormones coordinately induced genes encoding various functionally important enzymes, such as phenylalanine ammonia-lyase and cinnamate-4-hydroxylase, amongst others ‒ catalysing the biosynthesis of anthocyanins, phytoalexins, lignin and other defence-related secondary metabolites of the phenylpropanoid pathway^35,39^.

**Figure 7:**
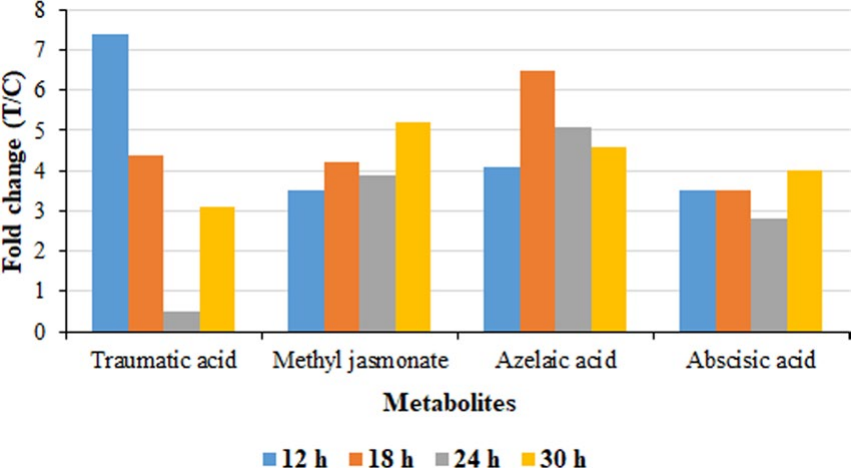
Relative quantification of significantly-accumulating plant hormones annotated in intracellular extracts and induced by LPS_*.a*._ treatment of sorghum cells. The graph shows the relative levels of each metabolite across different time points, expressed as fold changes, and computed from treated against control (C0 h) samples *i.e*. T/C, where fold change ≥1 represents significant accumulation, n = 9.

Another fatty acid-derived phytohormone, traumatic acid, which accumulated exclusively in the intracellular milieu, exhibited significant accumulation (Table 1). The 12-oxo-trans-10-dodecenoic acid-derived hormone, which displayed very high levels during the early stages (12–18 h) of the treatment (Fig. 7), is generally known as a wound hormone, due to high accumulation around wounded areas^40^. Synthesis of traumatic acid is commonly associated with abiotic stress factors^40^, however, the hormone was also identified as a resistance-inducing metabolite in barley for conferring resistance to *F. graminearum*^41^.

The hormones azelaic acid (AZA) and abscisic acid (ABA) were identified in high levels in both endo- and exometabolomes (Table 1). ROS accumulating in response to biotic stress (*e.g*. pathogen challenge^42,43^, can trigger the cleavage of fatty acids and yield products such as AZA, a signalling molecule also associated with systemic acquired resistance (SAR)^19,44^. Following pathogen infection, AZA ‒ as one of the signalling molecules - accumulates in petiole exudates and a small fraction translocates to distal tissue^45^. ABA is generally associated with defence responses to abiotic stress. However, the complex role of the hormone in plant immunity is continuously being uncovered^46^, with recently emerged insights into ABA’s role in plant–pathogen interactions as a positive or negative defence response regulator, depending on the phase/time of infection and nature of the pathogen^47^. Defence mechanisms such as stomatal closure, induced by ABA signalling in order to inhibit bacterial invasion, have been reported^48^. In some plant cell cultures, ABA has also been reported in the regulation of secondary metabolite biosynthesis^38^.

Metabolomic profiling of cultured cells revealed the intracellular induction of L-phenylalanine (phe) and L-tryptophan (trp), as well as the excretion thereof into the extracellular milieu (Table 1, S4 and S5). The role of these amino acids in plant defence includes functioning as regulators and precursors in various secondary metabolic pathways involved in MAMP-triggered immunity. Phe is a particularly important initiator/precursor molecule of the phenylpropanoid pathway and is also involved in SA biosynthesis, while trp is a major precursor in indolic secondary metabolite synthesis^48^. Moreover, the trp metabolic pathway is involved in defence responses in cereal crops, through the production of serotonin and conjugates^49^. The intracellular up-regulation of phe and trp (fold change>1) across the time points (Fig. S9) could be because of continuous requirement for precursors in the downstream synthesis of phe- and trp-derived metabolites, respectively. The presence of phenylpropanoids (and other related secondary metabolites) in the cell extracts indicates that these metabolic pathways were activated by the cells in response to ‘non-self’ perception^4^ of LPS_*B.a*_. as a MAMP. Similarly, the presence of trp and trp-derived metabolites such as indoles and serotonin conjugates, is also indicative of the active involvement of these pathways in LPS_*B.a*._-triggered immune responses.

**Figure 8:**
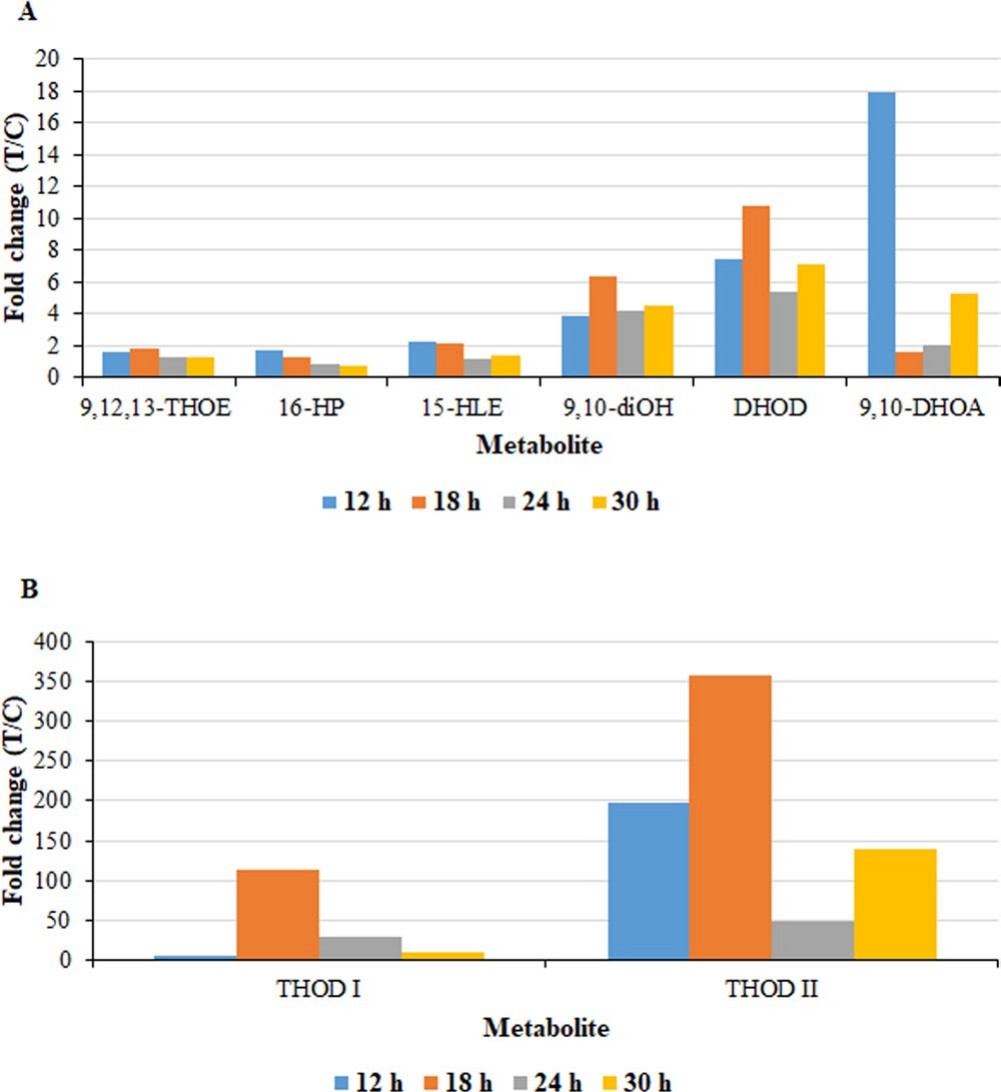
Relative quantification of fatty acids annotated in intracellular extracts and induced by LPS_*B.a*._ treatment of sorghum cells. The graph shows the relative levels of each metabolite across the time points, expressed as fold changes, and computed from treated against control (C0 h) *i.e*. T/C, where fold change ≥1 represents significant accumulation, n = 9. Graphical representation of the annotated fatty acids is divided into two graphs; **(A)** for reasonably high fold changes and **(B)** for very high fold changes. 9,12,13-THOE = 9,12,13-trihydroxy-10-octadecenoic acid; 15-HLE = 15-hydroxylinoleic acid; DHOD = dihydroxy-octadecadienoic acid; 16-HP = 16-hydroxypalmitate; 9,10-diOH = 9,10-dihydroxystearic acid; 9,10-DHOA = 9,10-dihydroxy-12-octadecenoic acid; THOD I = trihydroxy-octadecadienoic acid І; THOD II = trihydroxy-octadecadienoic acid ІІ.

LPS_*B.a*._ elicitation also triggered significant changes in lipidome components as infographically shown in Fig. 8A-B. These hydroxy fatty acids accumulated in the intracellular milieu of treated cells to varying degrees, with levels fluctuating across the time points. Assessing the quality of the endo- and exolipidome, some fatty acids and derivatives were detected in both intra- and extracellular extracts, whereas others were exclusive to the endolipidome (15-hydroxylinoleic acid, 9,10-dihydroxy-12-octadecenoic acid, 9,10-dihydroxystearic acid, trihydroxy-octadecadienoic acid І and 16-hydroxypalmitate) (Table 1). Fatty acids and derivatives thereof are crucial in basal immunity and gene-mediated resistance and contribute to inducing SAR^50,51^. Fatty acids have been linked to plant defence responses as enhancers of structural defence (cell membrane and cell wall), antimicrobial compounds, key players in plant defence signalling pathways (*e.g*. oxylipins) and to act as precursor substrates in the production of JA, an important mediator of plant defence^52,53^.

Significant accumulation of oxylipins, trihydroxy-octadecadienoic acid І, trihydroxy-octadecadienoic acid ІІ and 9,12,13-trihydroxy-10-octadecenoic acid, and the dihydroxy-oxylipin, 9,10-dihydroxy-12-octadecenoic acid, was observed in LPS_*B.a*__._-treated cells. Based on correlative data and experimental work, several trihydroxy-oxylipins have been shown to exhibit antimicrobial activity and establish resistance towards fungal and some bacterial pathogens, and to orchestrate defence responses in cereal plants like barley against powdery mildew and rice against rice blast disease^54,55^. For such metabolites to be significantly effective in inhibiting pathogen growth *in planta*, they should be available in adequate concentrations^54^. An interesting observation was the massive accumulation of the oxylipin, trihydroxy-octadecadienoic acid ІІ, of 356.7-fold at the 18 h time point in the cells (Table 1). An unsupervised colour-coded PCA score plot (Fig. S10) revealed the presence of this metabolite only in treated cells (intracellular extracts) and absent in the control (non-treated) samples, implying a *de nov*o biosynthesis of the metabolite as induced by LPS treatment. Trihydroxy-octadecadienoic acids such as 9,12,13-trihydroxy-10,15-octadecadienoic acid have been shown to possess antimicrobial activity towards both fungal and bacterial pathogens^52,55^.

The metabolomics results point to the importance of the oxylipin and fatty acid pathways in defence responses in sorghum cells, based on significant accumulation. However, the mechanisms by which oxylipins inhibit microbial growth through antimicrobial activity and to establish resistance in plants, is still largely undefined.

The pool size of induced metabolites may be determined by both the perceived stimulus and the metabolic activity occurring within the system^22,56^. Decrease in intracellular levels of secondary metabolites, accompanied by extracellular increases, may be due to the active translocation/secretion system of *S. bicolor*^57^ to the outside of the cell into the culture medium. This would functionally be similar to secretion into the apoplast/cell periphery in the tissue environment. Distribution of defence-related metabolites to the sites of early pathogen infection is crucial for the restriction of pathogen penetration and proliferation^58^, and a study on *A. thaliana* revealed the secretion of indolic glucosinolates upon LPS perception^21^.

Another possible explanation for the low intracellular levels of the phenylpropanoids and flavonoids in the cultured sorghum cells is the regulation of secondary metabolite levels by the cells, to avoid toxicity to the producing plant cell. In this regard, at certain levels some of the secondary metabolites become toxic to the producing cells, so the cells regulate levels in order to re-establish a form of homeostasis through interconversion, degradation, conjugation and secretion / translocation of potentially toxic metabolites^22,58^. Regulatory mechanisms by plants include transportation to the apoplast (*via* vesicles, simple diffusion, and transporter-mediated membrane transport) or specific organelles such as the vacuole, or other self-tolerance mechanisms^59^. Additionally, the decrease in intracellular phenolic levels could also be due to polymerisation of metabolites that act as monomers for polymers such as lignin in cell wall reinforcement, or due to transformation/conversion/conjugation into other defence-related metabolites^60,61^.

**Figure 9:**
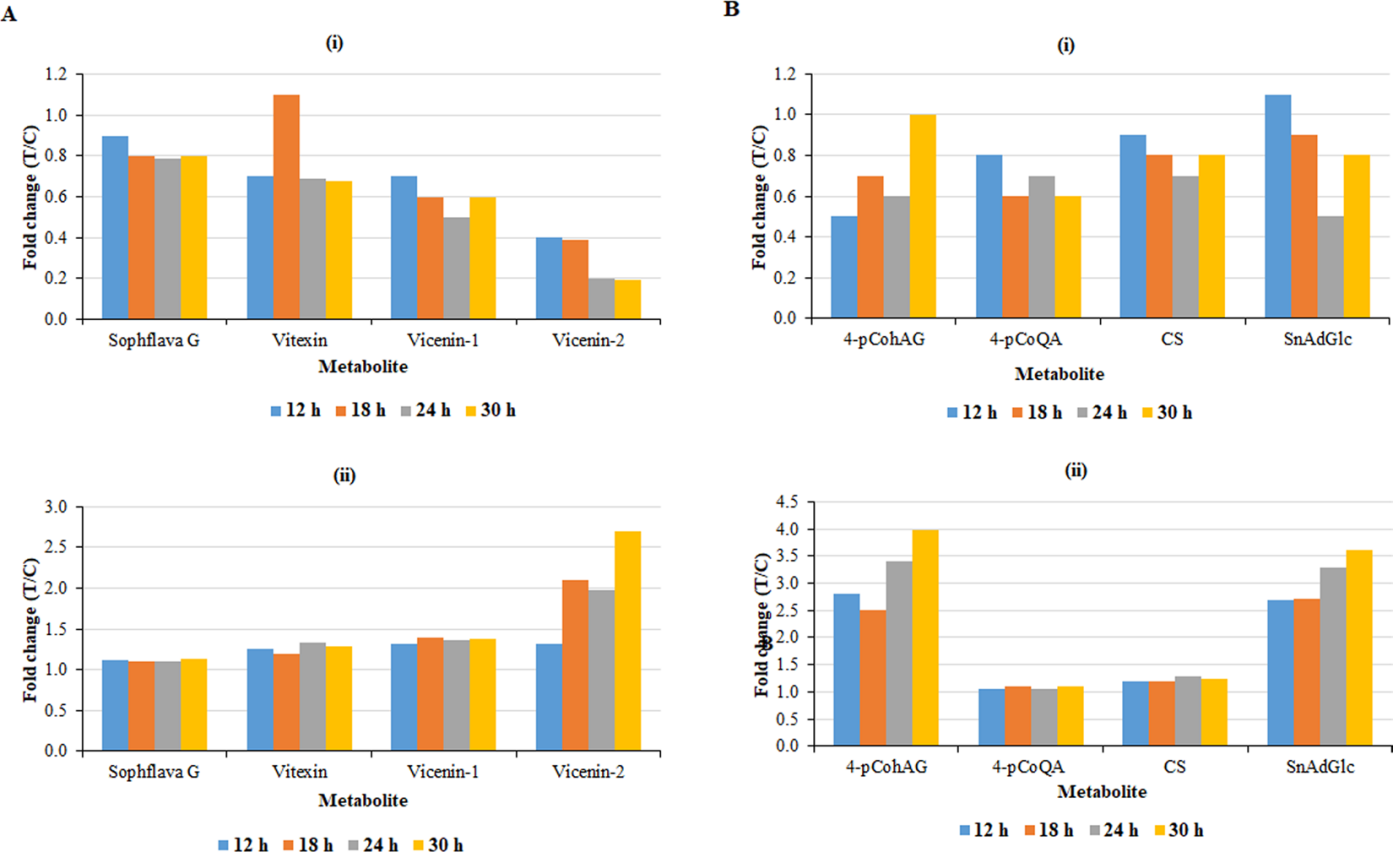
Relative quantification of some flavonoids (**A**) and hydroxycinnamic acids (**B**) annotated in (i) intracellular and (ii) extracellular extracts, and induced by LPS_*B.a*._ treatment of sorghum cells. The relative levels of each metabolite are expressed in fold changes, and computed from treated against control (C0 h) samples *i.e*. T/C, where fold change ≥1 represents significant accumulation, n = 9. Sophflava G = sophoraflavanone G; vicenin-1 = apigenin 6-C-xyloside-8-C-glucoside; vicenin-2 = apigenin-6,8-di-C-glucoside; vitexin = apigenin-8-C-glucoside 4-*p*CohAG = 4-coumaroyl-3-hydroxyagmatine; 4-*p*CoQA = 4-coumaroylquinic acid; CS = cinnamoylserotonin; SnAdGlc = sinapaldehyde glucoside.

Elicitation of cultured cells with LPS_*B.a*._ induced alterations in the phenylpropanoid and flavonoid metabolic pathways (Table 1, S4, and S5). Phenylpropanoid-based metabolites from a number of sub-pathways are known to play central role(s) in plant defence^62^. Relative quantification revealed that most of the metabolites arising from these pathways were generally associated with a decrease in levels (fold change <1), at most of the time points in the intracellular milieu (except for sinapoyl alcohol, Fig. 9A (i) **‒** flavonoids and Fig. 9B (i) ‒ hydroxycinnamic acids). Out of the fourteen phenolic compounds annotated, only sinapoyl alcohol was positively correlated to the LPS_*B.a*._ treatment, *i.e*. the other thirteen compounds were located/extracted from the bottom left quadrant of the OPLS-DA S-plot (*e.g*. Figure 6A and S6C). In parallel, the flavonoids and hydroxycinnamic acids (HCAs) displayed a general increase in the extracellular milieu (fold change>1) as seen in Fig. 9A (ii) and 9B (ii), respectively.

A focus on the identified flavonoids indicates that flavones (apigenin derivatives) were the dominant subgroup (Table 1). The biological functions of metabolites belonging to this subgroup in plant defence, such as exhibiting antimicrobial properties towards various pathogens, have been described in several plants^63^. The tetrahydroxyflavanone, sophoraflavanone G, also identified amongst the flavonoids, has been reported to possess antibacterial properties, with the mechanism of action including alteration of bacterial membrane fluidity^62,64^. The roles of the identified HCAs in plant defence include cell wall strengthening, precursors to defence metabolites and as antimicrobial compounds^22,65,66^. Sinapoyl alcohol, a syringyl lignin precursor, is an important metabolite in structural/mechanical defence. This is through lignification *i.e*. polymerisation of monolignols (precursors of lignin), resulting in reinforcement of the cell wall to become more resistant to pathogen-derived degrading enzymes and penetration by mycotoxins. In general, increased accumulation of this precursor associated with lignin deposition has been reported following pathogen challenge. Treatment with LPS_*B.a*._ resulted in high accumulation of sinapoyl alcohol, suggesting the importance of the metabolite in activation of structural defences in cultured cells^65–67^.

Other metabolites identified in this study and reported to be deployed in cell wall strengthening, include 4-coumaroyl-3-hydroxyagmatine, feruloylserotonin, sinapaldehyde glucoside, cinnamoylserotonin and dihydroconiferyl alcohol glucoside (a guaiacyl lignin monomer). In other studies these metabolites have also been identified as resistance-related compounds^41,60,67,68^. Additionally, 4-coumaroylquinic acid has been shown to confer resistance against a range of pathogens. Agmatine, which can conjugate with HCAs to yield HCA-amides, and important metabolites in cell wall strengthening and as phytoalexins^60,66,67^, displayed an increase and was found exclusively in the endometabolome.

The metabolic reprograming in sorghum cells as a result of LPS_*B.a*._ treatment also involved some alterations in flavin and indole metabolism. The metabolite profiles of treated cells were characterised by down- and up-regulation of riboflavin in the intra- and extracellular milieu, respectively (Table 1, S4 and S5). This is indicative of the secretion of the metabolite into the latter. Riboflavin has been identified as a defence response/systemic resistance-inducing metabolite in various plant species against bacterial, fungal and viral pathogens^23,69^. In rice, the metabolite was shown to induce defence responses against *Rhizoctonia solani* and *Pyricularia oryzae*^69^. The major role of the octadecanoid pathway in riboflavin-induced resistance and basal resistance, together with the link between riboflavin accumulation and increased lignification, was also highlighted earlier^36^.

Indolic derivatives (mostly indole acetyl derivatives), accumulated to varying levels in both the intra- and extracellular milieus (Table 1). A number of plant species and plant cell cultures have been reported to accumulate indolic derivatives as phytoanticipins, phytoalexins, precursors or as signalling molecules in response to pathogen and abiotic stress^21,47,49^. Indole-3-acetyl-myo-inositol, significantly detected in both the intra- and extracellular milieus of the sorghum cells, has been linked to plant resistance in barley^68^. The data obtained in this study therefore suggests defence-related roles for indole derivatives in sorghum cells. Moreover, the detection of both trp and indole-containing metabolites as discriminant ions / signatory biomarkers provides an insight into the activation of defences related to trp metabolism in response to LPS_*B.a*._ treatment. Together with the metabolites associated with the phenylpropanoid pathway, this indicates activation of an integrated and complementary network of chemical-based plant defences.

## Methods

### Bacterial cell culturing, LPS isolation and purification

An overnight bacterial culture of *B. andropogonis*, strain BD 256, obtained from the Plant Protection Institute, Agricultural Research Council (Pretoria, South Africa), was initially prepared by inoculating 600 mL nutrient broth medium (Merck, Johannesburg, RSA) and incubated overnight on a shaker at 28 °C and speed of 130 rpm. This was followed by inoculating large scale cultures (3 L of nutrient broth), with 200 mL of the overnight bacterial culture, under strict sterile conditions, and incubating at 30 °C on a rotating shaker at 100 rpm for 14 d. Several large-scale culturing cycles were repeated for larger quantities of cell mass for LPS isolation and purification. Harvesting of bacterial cells was performed by centrifugation at 13,000 × *g* at 4 °C for 20 min, followed by lyophilisation for 48 h before the LPS isolation and purification steps. LPS was extracted from the bacterial cell biomass using the hot-phenol water extraction protocol as previously described^70,71^. This involved lysing bacterial cells and partitioning of the LPS into the aqueous phase for maximum yield. For LPS purification, enzymatic digestion of the RNA that possibly co-extracted into the water phase, was executed. Dried extracts were dissolved in 30 mL (1: 40 *w/v*) distilled water and sequentially treated with RNase and proteinase K (Sigma-Aldrich, Steinheim, Germany) as previously described^14^. Following incubation, an equal volume of phenol was added in order to denature and remove the enzymes. Following vortexing, the emulsion was centrifuged at 10,000 × *g* for 15 min to obtain the hydrophilic LPS in the water phase. This was dialysed for 3 d against distilled water at 5 °C and then lyophilised.

### LPS-specific SDS-PAGE analysis and compositional analysis

The macromolecular nature of the purified LPS was evaluated by SDS-PAGE as previously described^72^. LPS from both *B. andropogonis* and *B. cepacia*^70,72^ were used for the analysis. Upon completion of electrophoresis, silver staining was performed for LPS visualisation^72^. For structural elucidation, an additional enzymatic treatment by RNase, DNase and Proteinase K (Roth, Germany) was performed in order to remove any contaminants. Subsequently a further step of purification by ultracentrifugation (100,000 ×*g*, 16 h, 4 °C) followed by size-exclusion chromatography on a Sephacryl S500 column was executed^10,14^. The LPS monosaccharide content was determined by analysis of the acetylated *O*-methyl glycoside derivatives obtained by treatment with HCl/MeOH (1.25 M, 85 °C, 24 h) followed by acetylation with acetic anhydride in pyridine (85 °C, 30 min). The absolute configuration was established through the evaluation of the *O*-octylglycoside derivatives^71,73^ of all sugars found in the LPS under investigation except for Rha3CMe. To define the sugar linkage pattern an aliquot of sample was methylated with CH_3_I, hydrolysed with trifluoroacetic acid (4 M, 100 °C, 4 h), carbonyl reduced with NaBD_4_ and acetylated with pyridine and acetic anhydride^74^. The total fatty acid content of intact LPS was determined by treating with HCl (4 M, 100 °C, 4 h) followed by a treatment with NaOH (5 M, 100 °C, 30 min). The pH was adjusted to reach slight acidity. After extraction in chloroform, fatty acids were methylated with diazomethane^75^. All chemical analyses were executed through the employment of gas-liquid chromatography (GLC-MS, Agilent Technologies 6850 A) equipped with a mass selective detector 5973 N and a Zebron ZB-5 capillary column.

### Isolation of lipid A and the OPS chain from *B. andropogonis* LPS

The pure LPS (20 mg) underwent a mild hydrolysis with acetate buffer solution (pH 4.4, 100 °C, 2 h). After centrifugation, the supernatant containing the OPS product, was collected and lyophilised (15 mg). A further purification step by size-exclusion chromatography on Sephacryl S100 followed by a Superdex 30 (GE Healthcare Life Sciences) was also executed to purify the product^10,11^. The precipitate, containing the LA, was collected and washed several times with a freshly prepared Bligh/Dyer mixture (chloroform/methanol/water, 2:2:1.8, v/v/v)^76^. The organic phases were pooled, dried and analysed by matrix-assisted laser desorption ionisation mass spectrometry (MALDI MS).

### NMR spectroscopy analysis

1D and 2D NMR spectra were executed on a Bruker 600 DRX instrument equipped with a cryo probe. The solvent was D_2_O. Spectra calibration was performed with internal acetone (*δ*_H_ 2.225 ppm, *δ*_C_ 31.45 ppm). The double-quantum filtered phase sensitive correlation spectroscopy (DQF-COSY) experiment was carried out by using data sets of 4096 × 256 points^77,78^. Total correlation spectroscopy (TOCSY) experiments were performed with spinlock times of 100 ms, using data sets (t1 × t2) of 4096 × 256 points. Rotating frame Overhauser enhancement spectroscopy (ROESY) and Nuclear Overhauser enhancement spectroscopy (NOESY) experiments were executed by using data sets (t1 × t2) of 4096 × 256 points and by using mixing times between 100 and 400 ms. In all homonuclear experiments the data matrix was zero-filled in both dimensions to give a matrix of 4 K × 2 K points and was resolution enhanced in both dimensions by a cosine-bell function before Fourier transformation. The determination of coupling constants was obtained by 2D phase sensitive DQF-COSY. Heteronuclear single quantum coherence (^1^H, ^13^C HSQC) and heteronuclear multiple bond correlation (^1^H, ^13^C HMBC) experiments were recorded in ^1^H-detection mode by single-quantum coherence with proton decoupling in the ^13^C domain using data sets of 2048 × 256 points. ^1^H, ^13^C HSQC was executed using sensitivity improvement and in the phase-sensitive mode using Echo/Antiecho gradient selection, with multiplicity editing during selection step^79^. The ^1^H, ^13^C HMBC experiment was optimised on long range coupling constants with low-pass *J* filter to suppress one-bond connectivity, using gradient pulses for selection. A delay of 60 ms was employed for the evolution of long range correlations. It was used at long range coupling constant value of 6 Hz. The data matrix in both heteronuclear experiments was extended to 2048 × 1024 points using forward linear prediction extrapolation^80^.

### MALDI mass spectrometry analysis

Reflectron MALDI-TOF MS was performed on an ABSCIEX TOF/TOF^TM^ 5800 Applied Biosystems mass spectrometer equipped with an Nd:YLF laser with a λ of 345 nm, a < 500-ps pulse length and a repetition rate of up to 1000 Hz. The LA fraction was dissolved in chloroform/methanol (1:1, *v/v*) as previously described^12,81^. The matrix was trihydroxyacetophenone dissolved in methanol/0.1% trifluoracetic acid/acetonitrile (7:2:1, *v/v/v*) at a concentration of 75 mg mL^−1^. Thereafter, 0.5 μL of the LA preparation and 0.5 μL of the matrix solution were deposited on the MALDI plate and dried at room temperature. Each spectrum was a result of the accumulation of 1500 laser shots^82^ and was recorded in negative polarity.

### Preparation and elicitation of *Sorghum bicolor* cell suspensions

Seeds of white sorghum, *Sorghum bicolor* (L.) Moench, used for the generation of cell suspension cultures were obtained from Agricol (Pretoria, South Africa). Cell suspensions were generated from friable callus initiated and maintained on Murashige and Skoog medium^83^ containing [3% (*w/v*) sucrose, pH 5.8] including MS vitamins and phytohormones [3 mg L^−1^ 2,4-dichlorophenoxyacetic acid (2,4-D) and 2.5 mg L^−1^ 1-naphthaleneacetic acid (NAA)] as described previously^84^.

To minimise unplanned variation in experimental plant material three independent biological repeats were conducted and analysed. To achieve this, cell suspensions were sub-cultured before every biological repeat to generate sufficient cell material for subsequent metabolomics analysis. Four days after subculture, synchronised cells from replicate flasks were first combined to ensure a homogeneous culture. Equal aliquots (25 mL) of the cell suspensions were then redistributed into sterile 50 mL Falcon tubes with three replicates for each condition. Cells were treated with LPS dissolved in the culture medium and negative controls received the equivalent volume medium. The time study to monitor the dynamics of the changing metabolomes included intervals of 0, 12, 18, 24 and 30 h, post-inoculation (h.p.i.). Following extraction and sample preparation, each sample was analysed in triplicate by UHPLC-MS generating n = 9, (3 independent biological replicates x 3 analytical replicates) as required for downstream metabolomic data analysis.

LPS_*B.a*._ (isolated and purified as described above) was prepared in MS medium at room temperature to give a 10 mg mL^−1^ stock solution. The final concentration of LPS_*B.a*._ for elicitation of the cells was 100 µg mL^−1^. The treated and non-treated cell suspensions were placed horizontally on an orbital shaker and incubated at 130 rpm and 25 °C. A time study was conducted to monitor the response of the cells to treatment over time compared to untreated cells at 0 h. Cells were harvested at 12, 18, 24 and 30 h.p.i. by means of centrifugation using a swinging bucket bench top centrifuge at 5100 rpm and 4 °C for 25 min. Pelleted cells and medium supernatants were separated and immediately stored at –80 °C until both intracellular and extracellular metabolite extraction steps could be performed. The above procedures were performed under strict sterile conditions.

### Metabolite extraction and sample preparation

Intracellular metabolites were extracted using 100% cold methanol. Initially the pelleted cells harvested by centrifugation were mixed with the extraction solvent in a ratio of 1:2 (*w/v*) and homogenised using an Ultra-Turrax homogeniser and sonication using a probe sonicator (Bandelin Sonopuls, Berlin, Germany) set at 55% power for 15 s, working at 4 °C. Samples were then prepared in a similar manner as previously described^2^, until reconstitution. For extracellular metabolites, the decanted supernatants (growth media) of each tube were first lyophilised and the obtained dried material weighed and kept at – 80 °C for reconstitution. The intra- and extracellular extracts were then reconstituted in 50% UHPLC-grade methanol (Romil Pure Chemistry, Cambridge, UK) in a 1:10 *m/w* ratio and filtered through 0.22 µm nylon filters into UHPLC glass vials fitted with 500 µL inserts.

### Ultra-high-performance liquid chromatography-high definition mass spectrometry (UHPLC-HDMS) analyses

Cell (intracellular) and medium (extracellular) extracts were analysed on an UHPLC-HDMS system: Waters Acquity UHPLC coupled in tandem to a SYNAPT G1 Q-TOF high definition mass spectrometer (Waters Corporation, Milford, MA, USA) *via* an electrospray ionisation (ESI) interface. An optimised UHPLC-HDMS method previously reported by us[2]^2^ was adopted. In short, the samples were separated on a reverse phase C18 column (150 mm × 2.1 mm ×1.8 µm – HSS T3, Waters Corporation, Milford, MA, USA) at 60 °C. A binary mobile phase solvent system consisting of solvent A: 0.1% formic acid in MilliQ water and solvent B: 0.1% formic acid in acetonitrile (Romil, Cambridge, UK), was used. The flow rate was set to 0.4 mL min^−1^ with gradient elution of the following conditions: 2% B maintained for 1 min, 95% B at 15 min ‒ maintained for 2 min, and a change back to the initial conditions at 18 min, followed by a 2 min equilibration time of the column. The total chromatographic run time was 20 min and the injection volume was 4 µL. The MS detector acquired data in both ESI positive and negative modes. The conditions were set as follows: capillary voltage of 2.5 kV, sampling cone at 30 V, extraction cone at 4 V, cone gas flow 50 L h^−1^, desolvation gas flow 550 L h^−1^, source temperature at 120 °C, desolvation temperature at 450 °C, scan time of 0.1 s and mass range of 100–1000 Da. Each sample was analysed in triplicate. To ensure that high mass accuracy of analytes (1–3 mDa) was acquired, online mass correction was done using a lock spray source: leucine encephalin (50 pg mL^−1^), [M + H]^+^ = 55.2766 and [M – H]^−^ = 554.2615. This reference calibrant was sampled every 15 s, producing an average intensity of 350 counts scan^−1^ in centroid mode. Sample analyses were randomised to provide stochastic stratification in sample acquisition to minimise measurement bias. To assess the reliability and reproducibility of the analyses, and for non-linear signal correction, quality control (QC) samples (prepared by combining equal volumes of all the individual samples) were used. The QC samples (6 injections) were analysed every 30–35 injections to monitor possible changes in the instrument response. QC runs were also performed at the beginning and end of each batch to ensure system equilibration. Blank samples (50% aqueous methanol) were randomly analysed to monitor background noise. To generate molecular fragment information for downstream structure elucidation and compound identification, a data-independent acquisition (DIA) method, namely MS^E^ was applied: the MS analyses were set to carry out non-fragmented as well as five fragmenting experiments simultaneously by applying alternating collision energy of 0 eV (unfragmented) and from 10 to 50 eV (fragmented).

### Data processing, multivariate data analyses and metabolite annotation

MassLynx^TM^ XS software was used to extract raw data, obtained from UHPLC-HDMS, and the MarkerLynx^TM^ software (Waters Corporation, Manchester, UK) for processing. Software parameters were the same for intra- and extracellular data, *i.e*. mass tolerance 0.01 Da, mass range 100–1000 Da, mass window 0.05 Da and a retention time (Rt) window of 0.20 min. The Rt range was 1.6–15 min for intracellular data and 1–13 min for extracellular data. The ion intensity threshold was set at 100 and 50 counts respectively for intracellular- and extracellular data. The data matrices obtained from MarkerLynx processing were exported into SIMCA 14 software (Umetrics, Umea, Sweden) for multivariate statistical analyses. The data were Pareto-scaled before principal component analysis (PCA) and orthogonal projection to latent structures discriminant analyses (OPLS-DA). Nonlinear iterative partial least squares (NIPALS) algorithm was a default SIMCA methodology used to handle the missing values. A seven-fold cross-validation (CV) was applied as a tuning and validating procedure in computing the models. This *k*-fold CV is the most common technique for model evaluation and selection in machine learning; where a dataset is iterated *k* times. In each round, the dataset is split into *k* parts: validation and training subsets. In other words, for a fixed number of components, the Y values of all individuals of each subset are predicted using a sub-model computed with the 6 other subsets, in this case of 7-fold CV. Results of this *k-*fold cross-validation procedure are summarised by the value of different quality parameters, such as R^2^ and Q^2^ metrics in this case. R^2^ indicates the goodness of fit (explained variation) and Q^2^ reflects the predictive ability of the model (predicted variation)^25^. Further metrics used to validate generated models are described in the results section. In addition to strong outliers detected through Hoteling’s T2-established normality (score space), moderate outliers were evaluated using the distance to the model in space X (DModX) plots permitted the assessment of moderate outliers. Furthermore, for variable selection, the extracted discriminating features were evaluated using variable importance in projection (VIP) scores. Metabolites were annotated as previously described^2,23,85^. The chemometrically selected variables were then structurally elucidated and putatively identified. Annotation was performed at level 2 of the Metabolomics Standards Initiative (MSI)^30^.

## Availability of data and material

The study design information, LC-MS raw data, analyses and data processing information, and the meta-data were deposited to the EMBL-EBI metabolomics repository (MetaboLights50), with the identifier MTBLS1204 (http://www.ebi.ac.uk/metabolights/MTBLS.1204).

## Supplementary information

Supplementary file.
