# Supplementary material for: Adaptive defence-related changes in the metabolome of Sorghum bicolor cells in response to lipopolysaccharides of the pathogen Burkholderia andropogonis
Source: Sci Rep. 2020 May 6;10:7626. doi: 10.1038/s41598-020-64186-y (PMC7203242; doi:10.1038/s41598-020-64186-y)
Supplement: Supplementary file 1 — Supplementary file. [file 41598_2020_64186_MOESM1_ESM.pdf]

Supplementary information

Adaptive defence-related changes in the metabolome of *Sorghum bicolor* cells in response to lipopolysaccharides of the pathogen *Burkholderia andropogonis*

Charity R. Mareya[1], Fidele Tugizimana[1], Flaviana Di Lorenzo[2], Alba Silipo[2], Lizelle A. Piater[1], Antonio Molinaro[2] and Ian A. Dubery[1]*.

[1]Research Centre for Plant Metabolomics, Department of Biochemistry, University of Johannesburg, Auckland Park, 2006, South Africa.
[2]Department of Chemical Sciences, University of Napoli Federico II, Complesso Universitario Monte Sant'Angelo, Via Cintia 4, 80126 Napoli, Italy.

*To whom correspondence should be addressed: Email: idubery@uj.ac.za

---

Additional file 1 - LPS sensing by plants

LPS from Gram-negative has long been recognised and a potent elicitor of the induction and modulation of plant defense responses[1]. Following the formulation of the 'zig-zag' model to explain features of plant innate immunity[2], LPS has been recognised as a microbe/pathogen-associated molecular pattern (M/PAMP) able to be sensed by plants and to initiate microbe/pathogen-triggered immunity (M/PTI). In the case of plants, the details about how LPSs from pathogenic bacteria was recognised is not known, and knowledge about the structural features contributing to MAMP activity is still minimal[3]. Up to 2015, details on potential receptors and recognition of particular epitopes was particularly lacking[4]. LA was reported to be perceived as a MAMP by some host plants of the Brassicaceae family[5] presumably *via* ligand-receptor interactions with putative patter-recognition receptors (PRRs) like the S-domain receptor-like kinase proteins[6]. A lectin S-domain receptor kinase from Arabidopsis, LORE (Lipo-Oligosaccharide-specific Reduced Elicitation), was reported to sense LPSs from *Xanthomonas* and *Pseudomonas* spp.[7]. However, a recent communication contradicted this and reported that perception in Arabidopsis was due to a medium-chain 3-hydroxy fatty acid, 3-OH-C10:0[8]. Reported immune responses of *A. thaliana* to LPSs from *Escherichia coli*, *Salmonella enterica*, or *Burkholderia* spp were not mediated by LORE[7], implying the involvement of other receptor(s) and/or non-receptor-based mechanisms. The true mechanisms of LPS sensing by plants therefore remain unknown.

1. Dow, M., Newman, M-A., & Von Roepenack, E. The induction and modulation of plant defense responses by bacterial lipopolysaccharides. *Annu. Rev. Phytopathol.* **38,** 241-261 (2000).

2. Jones, J.D.G. & Dangl, J.L. The plant immune system. *Nature* **444,** 323–329 (2006).

3. Newman, M.A., Sundelin, T., Nielsen, J.T., & Erbs, G. MAMP (microbe-associated molecular pattern) triggered immunity in plants. *Front Plant Sci.* **4,** 139 (2013).

4. Ranf S. Immune sensing of lipopolysaccharide in plants and animals: same but different. *PLoS Pathog.* **12(6),** e1005596 (2016).

5. Zeidler, D. *et al.*, Innate immunity in *Arabidopsis thaliana* : Lipopolysaccharides activate nitric oxide synthase and induce defense genes. *Proc Natl Acad Sci*. **101,** 15811–15816 (2006).

6. Sanabria, N.M., van Heerden, H., & Dubery, I.A. Molecular characterisation and regulation of a *Nicotiana tabacum* S-domain receptor-like kinase gene induced during an early rapid response to lipopolysaccharides. *Gene* **501,** 39–48 (2012).

7. Ranf, S. *et al.,* A lectin S-domain receptor kinase mediates lipopolysaccharide sensing in *Arabidopsis thaliana*. *Nat. Immunol.* **10,** 1038/ni.3124 (2015).

8. Kutschera, A. *et al.,* Bacterial medium-chain 3-hydroxy fatty acid metabolites trigger immunity in Arabidopsis plants. *Science* **364,** 178-182 10.1126/science.aau1279 (2019).

Additional file 2 - **Supplementary Tables and Figures**

Table S1. ^{1}H and ^{13}C (*italic*) chemical shifts (ppm) of the O-polysaccharide (OPS) chain fraction from *Burkholderia andropogonis* LPS.

Table S2. *Inter*-residual long-range connectivities from HMBC spectrum of the O-polysaccharide (OPS) chain fraction from *Burkholderia andropogonis* LPS.

Table S3. The main ion peaks in the MALDI-TOF MS spectrum reported in Fig. S2 and the proposed interpretation of the substituting fatty acids, phosphates (P) and aminoarabinoses (Ara4N) on the lipid A backbone. Some sodiated-adducts have been also reported.

Table S4. Annotated discriminatory metabolites from intracellular extracts of $LPS_{B.a.}$-treated *Sorghum bicolor* cultured cells, displaying the fold changes at different time points.

Table S5. Annotated discriminatory metabolites from extracellular extracts of $LPS_{B.a.}$-treated *Sorghum bicolor* cultured cells, displaying the fold changes at different time points.

Fig. S1. Zoom of the Total Correlation Spectroscopy (TOCSY) spectrum of the O-polysaccharide (OPS) chain fraction from *Burkholderia andropogonis* LPS. Key correlations are reported in the figure.

Fig. S2. Analysis of the Lipid A component of *B. andropogonis* LPS. (A) MALDI mass spectrometry analysis of the Lipid A component. (B) Lipid A structural representation.

Fig. S3. UHPLC-MS BPI chromatograms of methanolic extracellular extracts of sorghum cells treated with $LPS_{B.a.}$.

Fig. S4. PC analyses of the LC-MS ESI(+) data for intracellular sorghum cell extracts.

Fig. S5. PC analyses of the LC-MS data for extracellular sorghum cell extracts.

Fig. S6. Supervised multivariate analyses of the LC-MS ESI(+) data for intracellular extracts

Fig. S7. Supervised multivariate analyses of the LC-MS ESI(-) data for extracellular extract.

Fig. S8. Supervised multivariate analyses of the LC-MS ESI(+) data for extracellular extracts.

Fig. S9. Relative quantification of amino acids annotated in intracellular extracts and induced by LPS treatment of sorghum cells.

Fig. S10. PC analyses of LC-MS ESI(-) data of intracellular extracts from Sorghum cells.

Table S1. ^{1}H and ^{13}C (*italic*) chemical shifts (ppm) of the O-chain fraction from *Burkholderia andropogonis* LPS.

**Unit**	**1**	**2**	**3**	**4**	**5**	**6**
**A**	5.21	3.83	-	3.55	3.89	1.31
2-α-L-Rha*p*3*C*Me<br>-CH_3	*101.4*	*82.4*	*73.0*<br>*1.40/18.3*	*74.7*	*68.0*	*17.2*
**B**	5.04	4.14	3.90	3.59	3.91	1.31
3-α-L-Rha*p*	*102.5*	*70.1*	*77.8*	*71.6*	*69.3*	*16.9*
**C**	4.99	4.20	3.86	3.58	3.77	1.30
3-α-L-Rha*p*	*102.3*	*70.0*	*78.4*	*71.6*	69.6	*17.0*

Table S2. *Inter*-residual long-range connectivities from HMBC spectrum of the O-polysaccharide (OPS) chain fraction from *Burkholderia andropogonis* LPS.

**Sugar residue**	**Proton**	***δ ppm***	**HMBC correlation to atom**
**A** 2-α-L-Rha*p*3*C*Me	H-1	5.21	C-3 **B** (δ_C 77.8 ppm)
**B** 3-α-L-Rha*p*	H-1	5.04	C-3 **C** (δ_C 78.4 ppm)
**C** 3-α-L-Rha*p*	H-1	4.99	C-2 **A** (δ_C 82.4 ppm)

Table S3. The main ion peaks in the MALDI-TOF MS spectrum reported in Fig. S2 and the proposed interpretation of the substituting fatty acids, phosphates (P) and aminoarabinoses (Ara4N) on the lipid A backbone. Some sodiated-adducts have been also reported.

**Observed ion peaks (*m*/*z*)**	**Acyl substitution**	**Proposed fatty acid/phosphate/aminoarabinose composition**
1728.4	Penta-acyl	$HexN^2$ P^2 $Ara4N^2$ [14:0(3OH)][16:0(3OH)]^2 [14:0] Na^+
1670.5	Penta-acyl	$HexN^2$ P^2 [14:0(3OH)]^2[16:0(3OH)]^2 [14:0]
1597.3		$HexN^2$ P^2 Ara4N [14:0(3OH)][16:0(3OH)]^2 [14:0] Na^+
1575.3	Penta-acyl	$HexN^2$ P^2 Ara4N [14:0(3OH)][16:0(3OH)]^2 [14:0]
1495.4	Penta-acyl	$HexN^2$ P Ara4N [14:0(3OH)][16:0(3OH)]^2 [14:0]
1444.3	Tetra-acyl	$HexN^2$ P^2 [14:0(3OH)][16:0(3OH)]^2 [14:0]
1364.3	Tetra-acyl	$HexN^2$P [14:0(3OH)][16:0(3OH)]^2 [14:0]
1269.1	Tri-acyl	$HexN^2$ P Ara4N [16:0(3OH)]^2 [14:0]
1218.3	Tri-acyl	$HexN^2P^2$ [16:0(3OH)]^2 [14:0]
1138.1	Tri-acyl	$HexN^2$ P [16:0(3OH)]^2 [14:0]

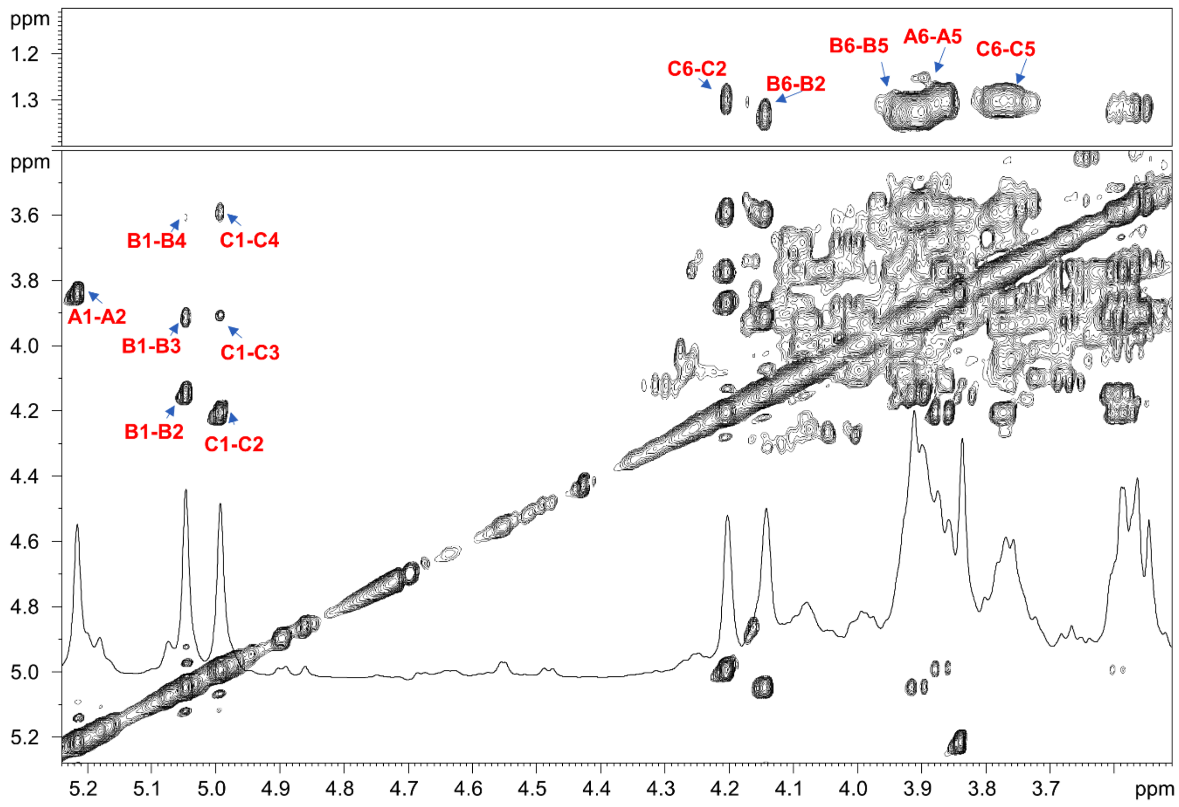

Fig. S1. Zoom of the TOCSY spectrum of the O-polysaccharide (OPS) chain fraction from *Burkholderia andropogonis* LPS. Key correlations are indicated in the figure.

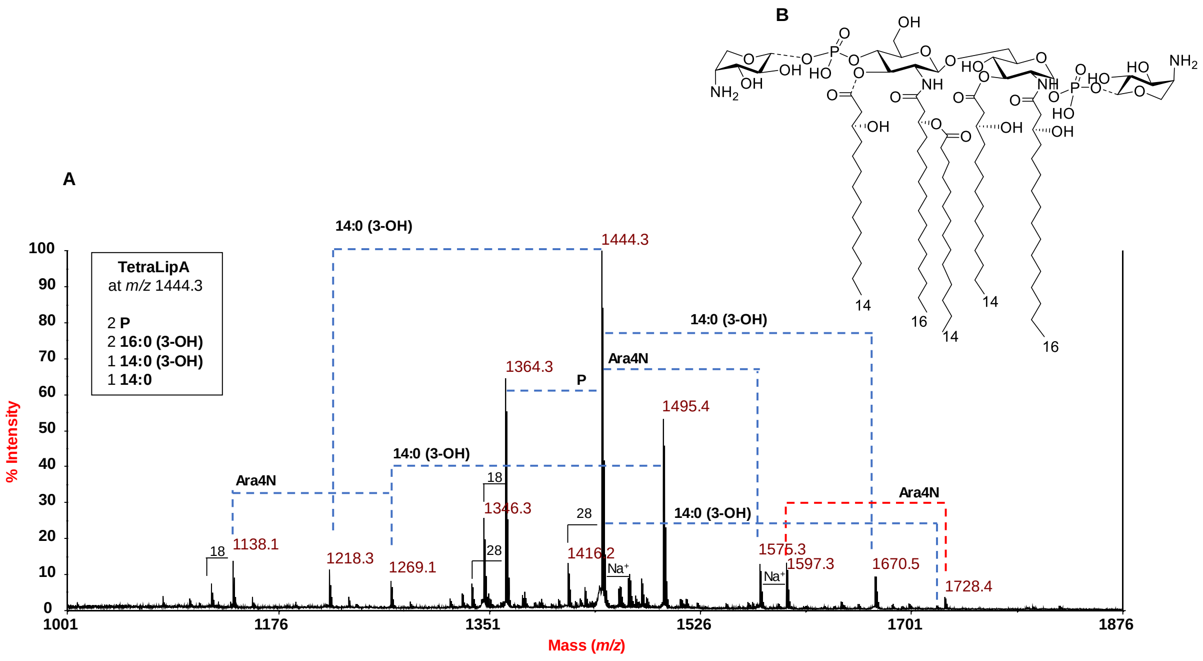

Fig. S2. Analysis of the lipid A component of *B. andropogonis* LPS. (A) MALDI mass spectrometry analysis of the lipid A component. (B) Lipid A structural representation. Structural analysis revealed that the lipid A is composed of a penta-acylated, 1,4'-*bis*-phosphorylated disaccharide backbone, which is further substituted by 4-amino-4-deoxy-L-arabinopyranose through a phosphodiester linkage, and fatty acid analysis revealed the presence of (*R*)-3-hydroxyhexadecanoic acid, (*R*)-3-hydroxytetradecanoic acid and tetradecanoic acid. Lipid A is regarded as a highly conserved component, differing amongst bacterial species and contributing to LPS stability.

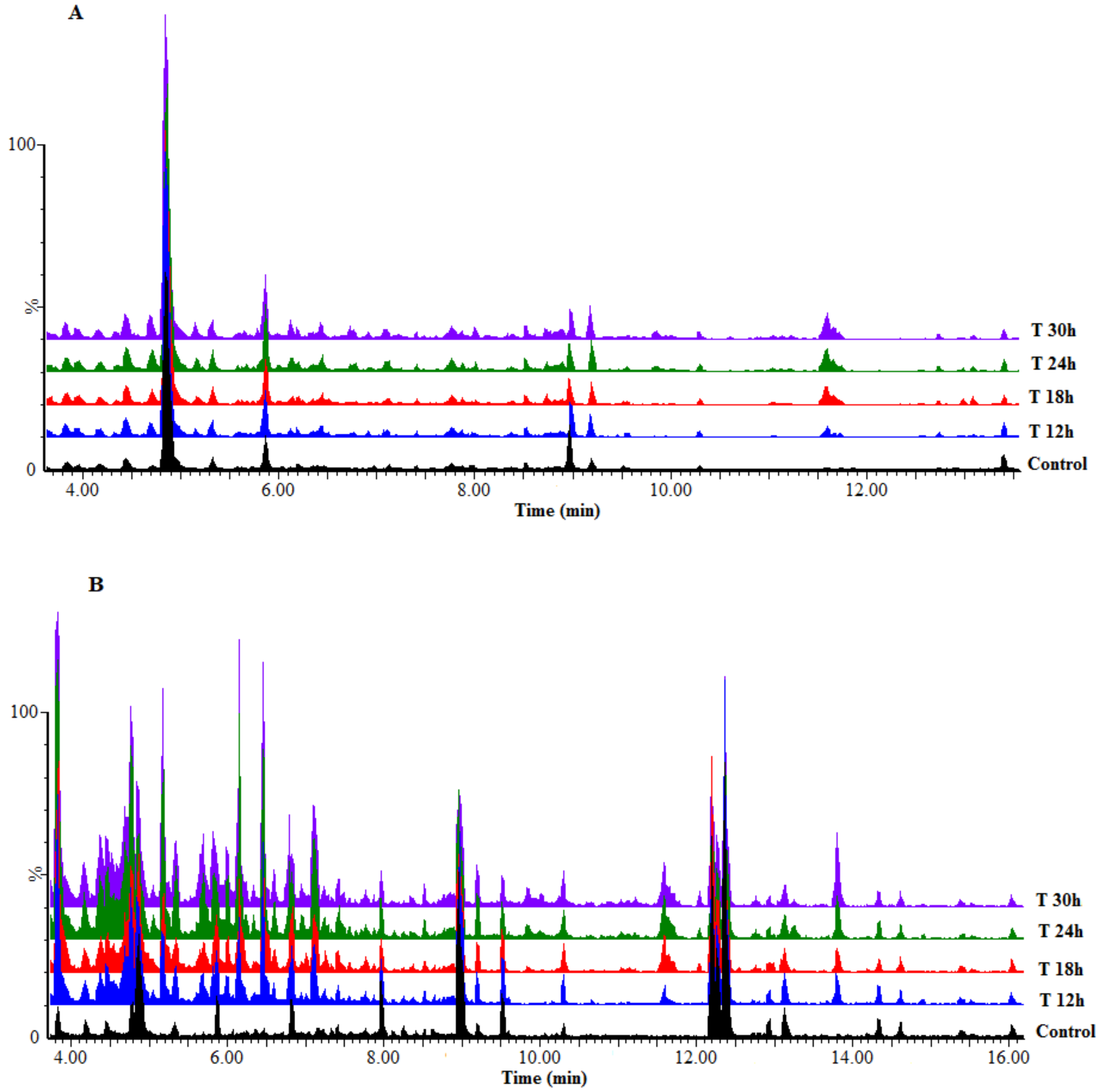

Fig. S3. UHPLC-MS BPI chromatograms of methanolic extracellular extracts of sorghum cells treated with LPS*B.a.*. (A): ESI(-) and (B): ESI(+). The chromatograms of a control (non-treated 0 h) *vs.* treated samples (12-30 h) display variation related to treatment- and time-related metabolic changes occurring in the cells due to LPS treatment. Equivalent MS chromatograms of methanolic intracellular extracts are presented in the main text as **Fig. 3.**

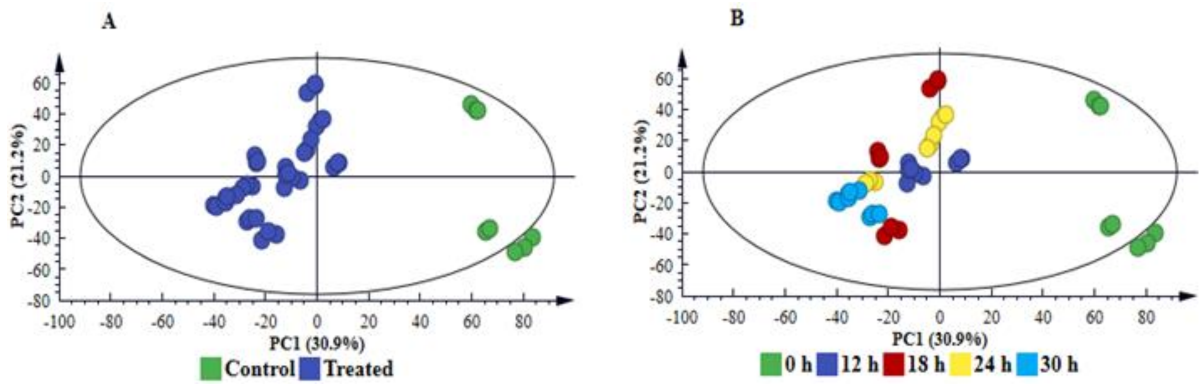

Fig. S4. PC analyses of the LC-MS ESI(+) data for <u>intracellular</u> sorghum cell extracts. The 5-component model explains 68.6% variations in Pareto-scaled data, X, and the amount of predicted variation by the model, according to cross-validation, is 57.3%. The first 2 PCs were used to generate the above scores plot of all data. (**A):** Clusters coloured based on condition *i.e.* non- *vs.* treated shows clear separation between treated and control (non-treated, 0 h) samples. (**B)** The same scores plot but coloured according to time. The equivalent analyses for **ESI(-)** data is presented in the main text, **Fig. 4**.

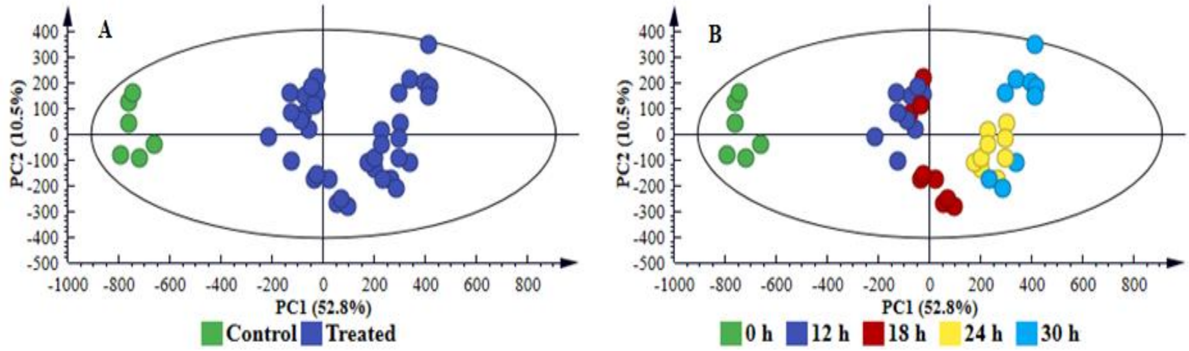

Fig. S5. PC analyses of the LC-MS data for <u>extracellular</u> sorghum cell extracts in ESI(+) mode. (A): The 3-component model explains 61.5% variation in Pareto-scaled data, X, and the amount of predicted variation by the model, according to cross-validation, is 52.8% **(B):** The 4-component model explains 78.6% variations in Pareto-scaled data, X, and the amount of predicted variation by the model, according to cross-validation, is 54.2%. The first 2 PCs were used to generate the above scores plot of all data. **A:** Clusters coloured based on condition *i.e.* non- *vs.* treated shows clear separation between treated and control (non-treated, 0 h) samples. **B** is the same scores plot but coloured according to time. The equivalent analyses for **ESI(-)** data is presented in the main text, **Fig. 5**.

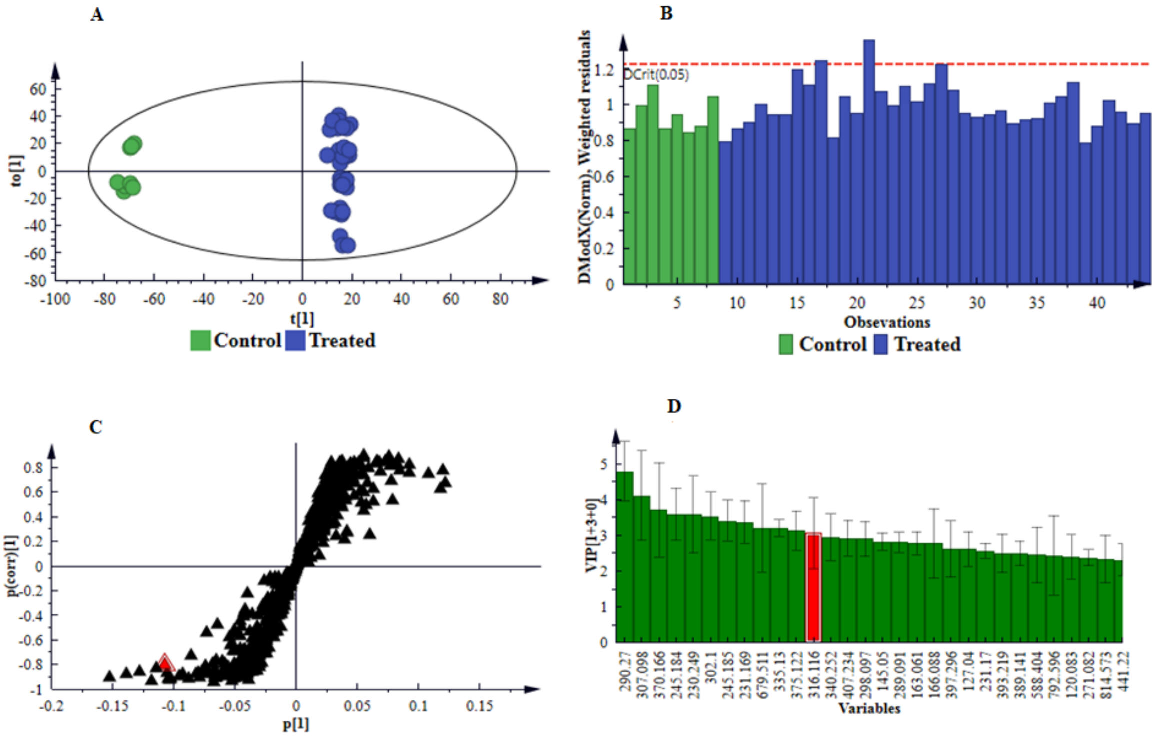

Fig. S6. Supervised multivariate analyses of the LC-MS ESI(+) data for <u>intracellular</u> extracts.

(A): Grouping of control (C0 h) *vs.* treated (all time points combined) as indicated by an OPLS-DA score plot. This model comprises 1 predictive component and 3 orthogonal components (R^2X= 58.2%, R^2Y= 99.6% and Q^2= 95.7%). **(B):** A distance to the model in space X (DModX) plot to detect outliers (above the dashed red line, Dcrit) in the OPLS-DA scores plot. **(C):** An OPLS-DA loadings S-plot displaying the discriminating features (ions) that explain the clustering (sample grouping) observed in the OPLS-DA scores plot, with the features in the top right quadrant positively correlated to the treatment and those in the bottom left quadrant negatively correlated to the treatment. **(D):** A VIP plot summarising the importance of some of the variables in the projection of the model with the *m/z* values and jackknife confidence intervals reflecting the variable stability indicated. A VIP value >1 is significant/important in the projection and increase in value indicates an increase in significance of the variable. The equivalent set of graphs for the **ESI(-)** data is presented in the main text as **Fig. 6**.

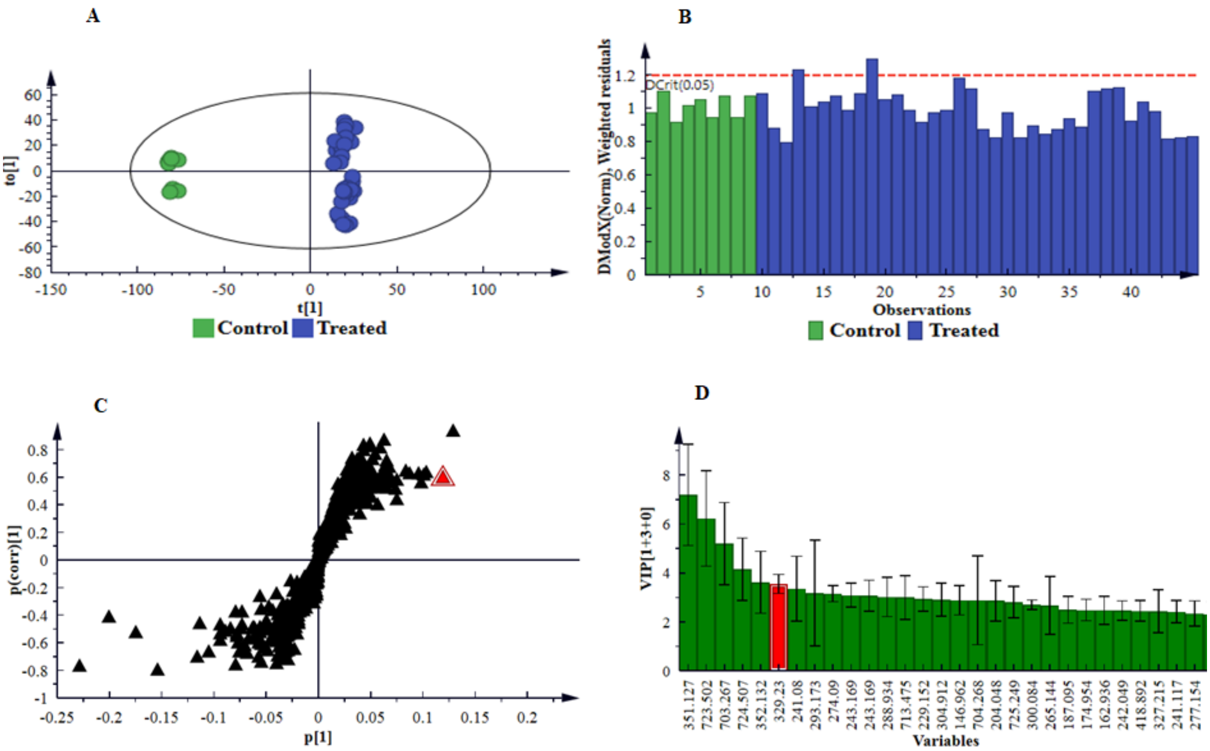

Fig. S7. Supervised multivariate analyses of the LC-MS ESI(-) data for <u>extracellular</u> extracts (excluding QCs). (A): Grouping of control (C0h) *vs.* treated (all time points combined) as indicated by an OPLS-DA score plot. This model comprises 1 predictive component and 2 orthogonal components (R^2X= 47.1%, R^2Y= 99.5% and Q^2= 94.0%). **(B):** A distance to the model in space X (DModX) to detect outliers (above the dashed red line, Dcrit) in the OPLS-DA scores plot. **(C):** An OPLS-DA loadings S-plot displaying the discriminating features (ions) that explain the clustering (sample grouping) observed in the OPLS-DA scores plot, with the features in the top right quadrant positively correlated to the treatment and those in the bottom left quadrant negatively correlated to the treatment. **(D):** A VIP plot summarising the importance of some of the variables in the projection of the model with the *m/z* values and jackknife confidence intervals reflecting the variable stability. A VIP value >1 indicates a significant variable in the complex analysis in comparing the difference between groups.

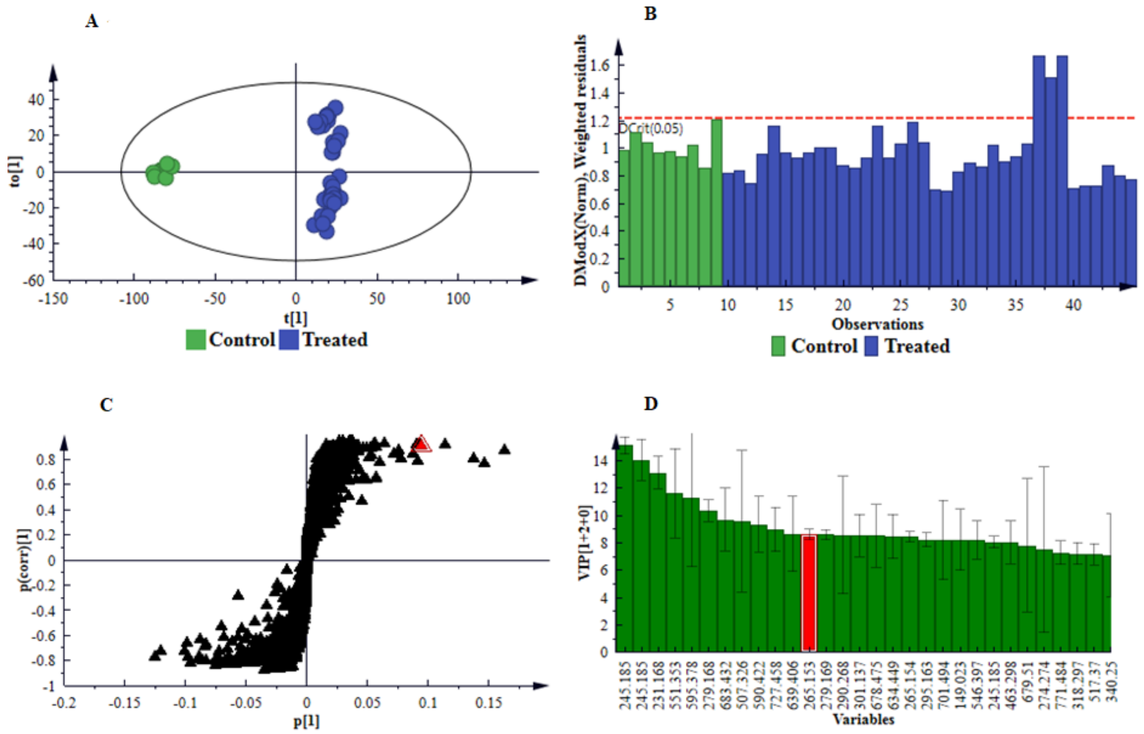

Fig. S8. Supervised multivariate analyses of the LC-MS ESI(+) data for extracellular extracts (excluding QCs). (A): Grouping of control (C0 h) *vs.* treated (all time points combined) as indicated by an OPLS-DA score plot. This model comprises 1 predictive component and 1 orthogonal components (R^2X= 53.8%, R^2Y= 99.1% and Q^2= 96.6%). **(B):** A distance to the model in space X (DModX) to detect outliers (above the dashed red line, Dcrit). **(C)** An OPLS-DA loadings S-plot displaying the discriminating features (ions) that explain the clustering (sample grouping) observed in the OPLS-DA scores plot, with the features in the top right quadrant positively correlated to the treatment and those in the bottom left quadrant negatively correlated to the treatment. **(D)** A VIP plot summarising the importance of some of the variables in the projection of the model with the *m/z* values and jackknife confidence intervals reflecting the variable stability. A VIP value >1 is significant/important in the projection and increase in value indicates an increase in significance of the variable.

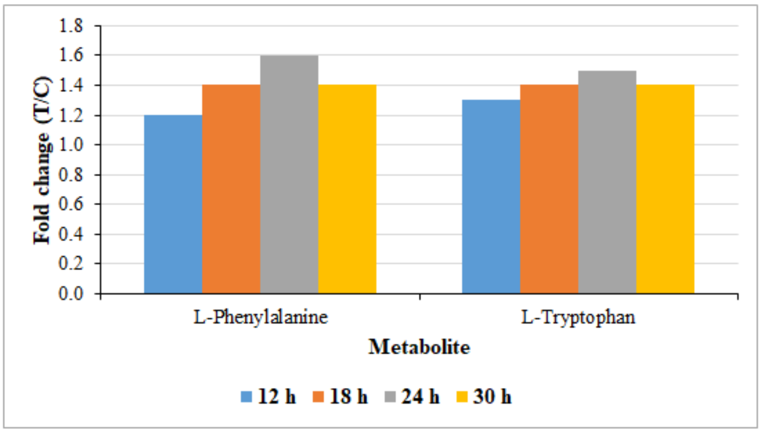

Fig. S9. Relative quantification of amino acids annotated in intracellular extracts and induced by LPS treatment of sorghum cells. The graph shows the relative levels of each metabolite across the time points, expressed as fold changes, and computed from treated against control (C0 h) *i.e.* T/C, where fold change > 1 represents significant accumulation.

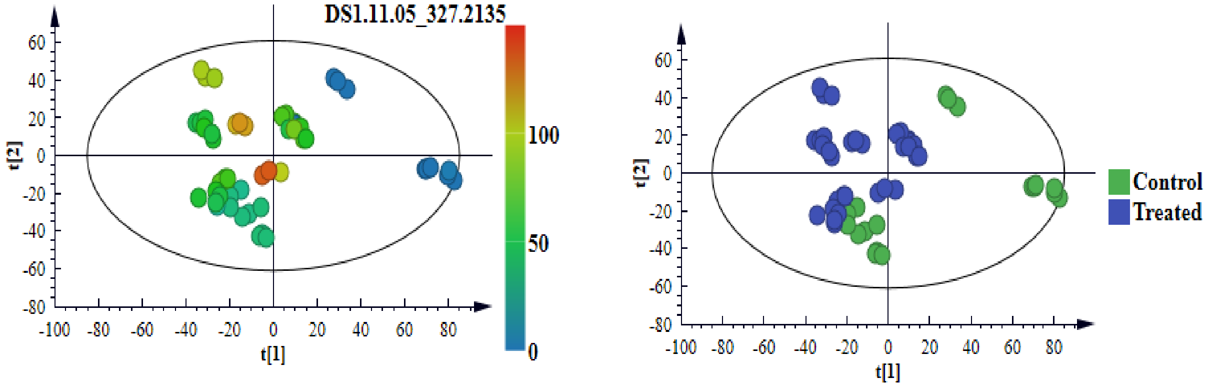

Fig. S10. PC analyses of LC-MS ESI(-) data of intracellular extracts from sorghum cells. (A) An unsupervised colour-coded PCA score plot displaying the presence/absence and intensity of the trihydroxy-octadecadienoic acid II phytoalexin across intracellular samples**. (B)** A similar corresponding PCA score plot, coloured coded based on condition (treated/control) to assist in indicating if samples belong to the control or treated group. The absence of the metabolite in non-treated (control) samples and presence in the treated samples indicate LPS-induced *de novo* biosynthesis.

Table S4. Annotated discriminatory metabolites from intracellular extracts of LPS$_{B.a.}$-treated *Sorghum bicolor* cultured cells, displaying the fold changes at different time points. The metabolites were annotated at MI-level 2 and had VIP scores > 1. Fold changes were obtained from OPLS-DA models computed of control 0 h *vs.* treated 12 h, 24 h and 30 h. (*Data for the 18 h time point is presented in the main text*).

Metabolites	*m/z*	Rt (min)	Adduct	Ion mode	Formula	C0 h *vs.* T12 h		C0 h *vs.* T24 h		C0 h *vs.* T30 h	
						p-value	Fold change	*p*-value	Fold change	*p*-value	Fold change
L-Phenylalanine	164.0686	1.84	$[M-H]^-$	neg	$C_9H_{11}NO_2$	0.015	1.2	1.28E-06	1.6	0.000	1.4
L-Tryptophan	203.0798	2.78	$[M-H]^-$	neg	$C_{11}H_{12}N_2O_2$	3.89E-06	1.3	7.12E-07	1.5	7.37E-05	1.4
15-Hydroxylinoleic acid	295.2253	14.29	$[M-H]^-$	neg	$C_{18}H_{32}O_3$	8.38E-05	2.3	0.533	1.2	0.119	1.4
Dihydroxyoctadecadienoic acid	311.2242	11.79	$[M-H]^-$	neg	$C_{18}H_{32}O_4$	0.001	7.4	1.54E-05	5.4	2.75E-10	7.1
9,10-Dihydroxy-12-octadecenoic acid	313.2354	12.67	$[M-H]^-$	neg	$C_{18}H_{34}O_4$	1.24E-11	17.9	0.386	2.0	7.36E-05	5.3
9,10-Dihydroxystearic acid	315.2511	13.51	$[M-H]^-$	neg	$C_{18}H_{36}O_4$	1.12E-06	3.9	4.20E-05	4.2	2.48E-09	4.5
Trihydroxyoctadecadienoic acid I	327.2149	9.72	$[M-H]^-$	neg	$C_{18}H_{32}O_5$	0.439777	5.2	0.072	29.4	0.381	10.5
Trihydroxyoctadecadienoic acid II	327.2135	11.05	$[M-H]^-$	neg	$C_{18}H_{32}O_5$	1.67E-06	197.9	3.16E-12	49.3	4.44E-06	138.8
9,12,13-Trihydroxy-10-octadecenoic acid	329.2327	9.60	$[M-H]^-$	neg	$C_{18}H_{34}O_5$	0.001	1.6	0.089	1.3	0.044	1.3
16-Hydroxypalmitate	273.2553	13.65	$[M+H]^+$	pos	$C_{16}H_{32}O_3$	0.037	1.7	0.702	0.9	0.253	0.7
Sophoraflavanone G	423.1821	4.42	$[M-H]^-$	neg	$C_{25}H_{28}O_6$	0.001	0.9	0.000	0.8	2.76E-05	0.8
Apigenin-8-C-glucoside (vitexin)	431.0974	5.58	$[M-H]^-$	neg	$C_{21}H_{20}O_{10}$	0.202	0.7	0.176	0.7	0.140	0.7
Apigenin-6-C-xyloside-8-C-glucoside (vicenin-1)	565.1545	4.94	$[M+H]^+$	pos	$C_{26}H_{28}O_{14}$	0.112	0.7	0.025	0.5	0.036	0.6
Apigenin-6,8-di-C-glucoside (vicenin-2)	595.1687	4.77	$[M+H]^+$	pos	$C_{27}H_{30}O_{15}$	0.101	0.4	0.030	0.2	0.022	0.2
Apigenin 7,4'-dimethyl ether	316.1157	8.29	$[M+H\_NH_3]^+$	pos	$C_{17}H_{14}O_5$	0.001	0.6	0.000	0.5	0.000	0.6
3',4'5-Trihydroxy-3,7-dimethoxyflavone	367.0221	3.90	$[M-H]^-$	neg	$C_{17}H_{20}O_9$	0.068	0.6	0.003	0.6	0.067	0.8
4-Coumaroyl-3-hydroxyagmatine	291.1471	5.72	$[M-H]^-$	neg	$C_{14}H_{20}N_4O_3$	0.008	0.5	0.001	0.6	0.767	1.0
4-Coumaroylquinic acid	337.1474	1.77	$[M-H]^-$	neg	$C_{16}H_{18}O_8$	7.02E-05	0.8	3.73E-07	0.7	3.62E-09	0.6
Cinnamoylserotonin	351.1251	2.43	$[M-H\_HCOOH]^-$	neg	$C_{19}H_{18}N_2O_2$	0.000	0.9	5.97E-07	0.7	1.74E-06	0.8
Feruloylserotonin	351.1266	2.86	$[M-H]^-$	neg	$C_{20}H_{20}N_2O_4$	4.60E-06	0.8	0.985	1.0	0.527	1.6
Sinapaldehyde glucoside	369.1199	3.61	$[M-H]^-$	neg	$C_{17}H_{22}O_9$	0.781	1.1	0.040	0.5	0.069	0.8
1-O-Coumaroyl-beta-D-glucose	371.0957	4.94	$[M-H\_NaNa]^-$	neg	$C_{15}H_{18}O_8$	1.97E-07	0.8	1.25E-12	0.5	1.21E-12	0.5
Sinapyl alcohol	209.0764	6.72	$[M-H]^-$	neg	$C_{11}H_{14}O_4$	1.31E-06	3.5	6.93E-07	5.3	2.87E-05	3.6
Dihydroconiferyl alcohol glucoside	413.1422	3.27	$[M+H\_HCOONa]^+$	pos	$C_{16}H_{24}O_8$	0.006	0.7	0.001	0.5	0.006	0.7
Indole-3-butyric acid	272.0893	2.84	$[M+H\_HCOONa]^+$	pos	$C_{12}H_{13}NO_2$	0.000	0.5	8.13E-06	0.6	5.71E-07	0.5
N(6)-[(Indol-3-yl)acetyl]-L-lysine	304.1667	4.20	$[M+H]^+$	pos	$C_{16}H_{21}N_3O_3$	1.11E-07	5.2	1.42E-06	4.7	1.14E-09	6.7

Indole-3-acetyl-myo-inositol	353.1348	2.44	$[M-H\_NH_3]^-$	neg	$C_{16}H_{19}NO_7$	0.427	0.9	0.624572	0.9	0.114	0.8
Indole-3-acetyl-beta-1-D-glucoside	382.1121	3.93	$[M-H\_HCOOH]^-$	neg	$C_{16}H_{19}NO_7$	0.074	0.7	0.009	0.4	0.064	0.7
6-Hydroxy-indole-3-acetyl-valine	291.1294	3.89	$[M+H]^+$	pos	$C_{15}H_{17}N_2O_4$	0.056	0.3	0.023	0.2	0.136	0.5
Traumatic acid	297.1291	3.90	$[M+H\_HCOONa]^+$	pos	$C_{12}H_{20}O_4$	0.000	7.4	0.524	0.5	0.189	3.1
(9R,13R)-1a,1b-Dihomo-jasmonic acid	239.1638	12.19	$[M+H]^+$	pos	$C_{14}H_{22}O_3$	0.034	0.8	0.750	1.0	0.001	0.7
Zeatin-7-beta-D-glucoside	397.1826	6.73	$[M-H\_NH_3]^-$	neg	$C_{16}H_{23}N_5O_6$	0.079	0.7	0.018	0.7	0.084	0.8
Zeatin	220.1197	2.15	$[M+H]^+$	pos	$C_{10}H_{13}N_5O$	6.94E-05	0.6	4.39E-06	0.5	7.74E-06	0.5
Methyl jasmonate	247.1298	2.52	$[M+H\_Na]^+$	pos	$C_{13}H_{20}O_3$	1.31E-08	3.4	5.94E-11	3.9	1.31E-11	5.2
Dihydrozeatin riboside	354.1769	6.20	$[M+H]^+$	pos	$C_{15}H_{23}N_5O_5$	0.001	0.4	0.001	0.4	0.006	0.5
Zeatin riboside	374.1463	5.39	$[M+H\_Na]^+$	pos	$C_{15}H_{21}N_5O_5$	0.001	0.4	0.000	0.2	4.24E-05	0.1
Azelaic acid	187.0935	6.74	$[M-H]^-$	neg	$C_9H_{16}O_4$	2.45E-17	4.1	1.64E-17	5.1	2.29E-12	4.6
Abscisic acid	265.1552	3.35	$[M+H]^+$	pos	$C_{15}H_{20}O_4$	2.55E-08	3.5	1.18E-09	2.8	2.34E-09	4.0
Agmatine	173.0787	5.52	$[M-H\_NaNa]^-$	neg	$C_5H_{14}N_4$	1.74E-06	1.3	0.000	1.2	0.001	1.7
Riboflavin	377.1476	4.49	$[M+H]^+$	pos	$C_{17}H_{20}N_4O_6$	0.241135	0.8	0.000	0.3	0.001	0.4

Table S5. Annotated discriminatory metabolites from extracellular extracts of $LPS_{B.a.}$-treated *Sorghum bicolor* cultured cells, displaying the fold changes at different time points. The metabolites were annotated at MSI-level 2 and had VIP scores > 1. Fold changes were obtained from OPLS-DA models computed of control 0 h *vs.* treated 12 h, 24 h and 30 h. (*Data for the 18 h time point is presented in the main text*).

Metabolites	*m/z*	Rt (min)	Adduct	Ion mode	Formula	C0 h *vs.* T12 h		C0 h *vs.* T24 h		C0 h *vs.* T30 h	
						p-value	Fold change	*p*-value	Fold change	*p*-value	Fold change
L-Phenylalanine	164.0686	1.84	$[M-H]^-$	neg	$C_9H_{11}NO_2$	3.07E-11	2.7	1.04E-12	2.6	5.87E-12	2.6
L-Tryptophan	203.0798	2.78	$[M-H]^-$	neg	$C_{11}H_{12}N_2O_2$	0.001	1.4	0.000	1.4	2.14E-05	1.5
Dihydroxyoctadecadienoic acid	311.2242	11.79	$[M-H]^-$	neg	$C_{18}H_{32}O_4$	0.110	5.2	0.002	4.9	0.012	4.2
Trihydroxyoctadecadienoic acid II	327.2135	11.05	$[M-H]^-$	neg	$C_{18}H_{32}O_5$	0.001	22.7	0.158	52.4	4.02E-11	53.4
9,12,13-Trihydroxy-10-octadecenoic acid	329.2327	9.60	$[M-H]^-$	neg	$C_{18}H_{34}O_5$	1.13E-08	9.2	2.82E-09	8.4	1.13E-10	6.8
Sophoraflavanone G	423.1821	4.42	$[M-H]^-$	neg	$C_{25}H_{28}O_6$	0.030	1.1	0.054	1.1	0.028	1.1
Apigenin-8-C-glucoside (vitexin)	431.0974	5.58	$[M-H]^-$	neg	$C_{21}H_{20}O_{10}$	0.129	1.3	0.057	1.3	0.079	1.3
Apigenin-6-C-xyloside-8-C-glucoside (vicenin-1)	565.1545	4.94	$[M+H]^+$	pos	$C_{26}H_{28}O_{14}$	0.006	1.3	0.002	1.4	0.002	1.4
Apigenin-6,8-di-C-glucoside (vicenin-2)	595.1687	4.77	$[M+H]^+$	pos	$C_{27}H_{30}O_{15}$	0.873	1.3	0.159	2.0	0.014	2.70
4-Coumaroyl-3-hydroxyagmatine	291.1471	5.72	$[M-H]^-$	neg	$C_{14}H_{20}N_4O_3$	8.17E-05	2.8	4.32E-06	3.4	4.25E-07	4.0
4-Coumaroylquinic acid	337.1474	1.77	$[M-H]^-$	neg	$C_{16}H_{18}O_8$	0.138475	1.1	0.129	1.1	0.013	1.1
Cinnamoylserotonin	351.1251	2.43	$[M-H\_HCOOH]^-$	neg	$C_{19}H_{18}N_2O_2$	2.81E-05	1.2	1.28E-06	1.3	7.71E-06	1.2
Feruloylserotonin	351.1266	2.86	$[M-H]^-$	neg	$C_{20}H_{20}N_2O_4$	0.738	1.3	0.520	1.6	0.185	3.1
Sinapaldehyde glucoside	369.1199	3.61	$[M-H]^-$	neg	$C_{17}H_{22}O_9$	1.54E-06	2.7	2.61E-08	3.3	3.73E-09	3.6
1-O-Coumaroyl-beta-D-glucose	371.0957	4.94	$[M-H\_NaNa]^-$	neg	$C_{15}H_{18}O_8$	0.001	1.8	6.03E-05	1.9	8.81E-07	2.3
Sinapyl alcohol	209.0764	6.72	$[M-H]^-$	neg	$C_{11}H_{14}O_4$	5.80E-12	3.5	1.64E-10	2.8	1.73E-06	2.2
Indole-3-acetyl-myo-inositol	353.1348	2.44	$[M-H\_NH_3]^-$	neg	$C_{16}H_{19}NO_7$	0.086	2.2	0.007	2.6	0.001	2.6
Indole-3-acetyl-beta-1-D-glucoside	382.1121	3.93	$[M-H\_HCOOH]^-$	neg	$C_{16}H_{19}NO_7$	0.941	0.9	0.494	0.9	0.407	0.9
6-Hydroxy-indole-3-acetyl-valine	291.1294	3.89	$[M+H]^+$	pos	$C_{15}H_{17}N_2O_4$	0.000	2.7	3.67E-05	3.0	1.23E-05	3.3
Azelaic acid	187.0935	6.74	$[M-H]^-$	neg	$C_9H_{16}O_4$	2.04E-17	5.5	4.47E-15	4.1	6.58E-09	3.6
Abscisic acid	265.1552	3.35	$[M+H]^+$	pos	$C_{15}H_{20}O_4$	1.26E-13	16.2	1.24E-15	23.7	3.71E-15	22.9
Riboflavin	377.1476	4.49	$[M+H]^+$	pos	$C_{17}H_{20}N_4O_6$	2.54E-06	4.2	5.27E-08	7.1	2.07E-10	7.4
